# In Situ Forming of Nitric Oxide and Electric Stimulus for Nerve Therapy by Wireless Chargeable Gold Yarn‐Dynamos

**DOI:** 10.1002/advs.202303566

**Published:** 2023-10-22

**Authors:** Min‐Ren Chiang, Ya‐Hui Lin, Wei‐Jie Zhao, Hsiu‐Ching Liu, Ru‐Siou Hsu, Tsu‐Chin Chou, Tsai‐Te Lu, I‐Chi Lee, Lun‐De Liao, Shih‐Hwa Chiou, Li‐An Chu, Shang‐Hsiu Hu

**Affiliations:** ^1^ Department of Biomedical Engineering and Environmental Sciences National Tsing Hua University Hsinchu 300044 Taiwan; ^2^ Brain Research Center National Tsing Hua University Hsinchu 300044 Taiwan; ^3^ Department of Chemistry Stanford University Stanford CA 94305 USA; ^4^ Institute of Analytical and Environmental Sciences National Tsing Hua University Hsinchu 300044 Taiwan; ^5^ Institute of Biomedical Engineering National Tsing Hua University Hsinchu 300044 Taiwan; ^6^ Department of Chemistry National Tsing Hua University Hsinchu 300044 Taiwan; ^7^ Department of Chemistry Chung Yuan Christian University Taoyuan 320314 Taiwan; ^8^ Institute of Biomedical Engineering and Nanomedicine National Health Research Institutes Miaoli County 35053 Taiwan; ^9^ Institute of Pharmacology College of Medicine National Yang Ming Chiao Tung University Taipei 112304 Taiwan; ^10^ Department of Medical Research Taipei Veterans General Hospital Taipei 112201 Taiwan

**Keywords:** gas therapy, gold nanoparticles, nerve regeneration, nitric oxide, wireless charging

## Abstract

Endogenous signals, namely nitric oxide (NO) and electrons, play a crucial role in regulating cell fate as well as the vascular and neuronal systems. Unfortunately, utilizing NO and electrical stimulation in clinical settings can be challenging due to NO's short half‐life and the invasive electrodes required for electrical stimulation. Additionally, there is a lack of tools to spatiotemporally control gas release and electrical stimulation. To address these issues, an “electromagnetic messenger” approach that employs on‐demand high‐frequency magnetic field (HFMF) to trigger NO release and electrical stimulation for restoring brain function in cases of traumatic brain injury is introduced. The system comprises a NO donor (poly(S‐nitrosoglutathione), pGSNO)‐conjugated on a gold yarn‐dynamos (GY) and embedded in an implantable silk in a microneedle. When subjected to HFMF, conductive GY induces eddy currents that stimulate the release of NO from pGSNO. This process significantly enhances neural stem cell (NSC) synapses' differentiation and growth. The combined strategy of using NO and electrical stimulation to inhibit inflammation, angiogenesis, and neuronal interrogation in traumatic brain injury is demonstrated in vivo.

## Graphical abstract

An “electromagnetic messenger” approach utilizing high‐frequency magnetic fields (HFMF) to trigger nitric oxide (NO) release and electrical stimulation is developed for restoring brain function in traumatic brain injury. Upon exposure to HFMF, in vivo experiments demonstrated the combined strategy's effectiveness in inhibiting inflammation, angiogenesis, and neuronal interrogation in traumatic brain injury.

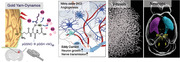

## 1 Introduction

Traumatic brain injury (TBI) is a major public health problem, responsible for a large number of injury‐related deaths and severe long‐term disability worldwide. It is estimated that as many as 50 million people suffer from a TBI each year, which can have devastating consequences for physical, cognitive and behavioral function.^[^ ^1^ ^]^ The initial injury occurs due to initial mechanical force, which can lead to direct damage to glial cells, vessels and neurons. This injury triggers a cascade of inflammatory events, including the infiltration of microglia/macrophages and activated astrocytes. This secondary injury involves neuronal and glial cell death, leading to impair brain functions and severe glial scar, hindering recovery from TBI.^[^ ^2^, ^3^, ^4^, ^5^ ^]^ After TBI, Cerebral atrophy, a common complication, is important for the recovery of brain tissue, manifested in the motor and sensory cortex, resulting in an impeded return of normal brain function.^[^ ^6^ ^]^ Despite recent advances in neuroscience, the difficulties in TBI treatments still stems from the heterogeneity and high complexity of TBI, and effective treatments for recovery from TBI remain lacking.^[^ ^7^, ^8^ ^]^

Nitric oxide (NO) is an important signaling molecule in the body, regulating a wide range of physiological functions such as blood flow, immune response and neural activity.^[^ ^9^, ^10^ ^]^ While overproduction may lead to cellular damage, therapeutic application of NO can stimulate angiogenesis by activating various growth factors while the concentration was lower than 400 nM.^[^ ^11^, ^12^ ^]^ In this regard, NO can stimulate the release of inflammatory mediators, such as interleukin‐1β, histamine, PAF, and TNF, making it a promising therapeutic agent.^[^ ^13^ ^]^ Numerous topical NO‐releasing biomaterials have been developed to accelerate wound healing.^[^ ^14^, ^15^, ^16^ ^]^ In the neuron system, NO acts as a neurotransmitter that activates the cyclic guanosine monophosphate (cGMP) pathway, which is essential for neurite growth and synapse remodeling after injury, facilitating to promote neuron regeneration.^[^ ^17^, ^18^, ^19^, ^20^ ^]^ Furthermore, NO affects synaptic plasticity, neurotransmission, and neurosecretion.^[^ ^21^, ^22^, ^23^, ^24^, ^25^ ^]^ For instance, Jiang et al. developed NO release nanoparticles effectively repairing spinal cord injury.^[^ ^21^ ^]^ However, the difficulties in translation still stem from the spatiotemporal control of NO release owing to the preservation issue and low diffusion efficacy of NO under physiological conditions.^[^ ^26^ ^]^

To address the issue, one possible solution is to combine the advantages of modular flexibility of delivery systems with the prodrug of nitric oxide (NO).^[^ ^27^, ^28^ ^]^ Various exogenous NO donors have been developed, such as organic nitrates, N‐diazeniumdiolates, S‐nitrosothiols, and metal‐nitrosyl complexes,^[^ ^29^, ^30^, ^31^ ^]^ which can be delivered using polymeric delivery systems to enhance their stability for sustained NO release.^[^ ^34^, ^35^, ^36^, ^37^, ^38^ ^]^ Certain delivery systems can also enable on‐demand NO release through physical stimuli such as enzymes, heat, light, ultrasound, and magnetic fields.^[^ ^39^, ^40^, ^41^, ^42^, ^43^, ^44^ ^]^ For instance, upconversion nanoparticles loaded with S‐nitrosocysteine can trigger NO release through the light‐induced cleavage of an S‐NO bond using near‐infrared light, while sono‐responsive NO‐donor‐conjugated delivery systems can trigger NO release via ultrasound‐induced free radicals.^[^ ^45^, ^46^ ^]^ However, the penetration of NIR light and the attenuation of ultrasound by tissue can limit the effectiveness of these methods in deep organs.^[^ ^47^ ^]^

Several non‐invasive neuromodulation techniques have been clinically employed to treat brain disorders, such as electrical deep brain stimulation (DBS), transcranial direct current stimulation (tDCS), and transcranial magnetic stimulation (TMS).^[^ ^48^, ^49^ ^]^ Electromagnetic field (EMF) has also been studied extensively for its therapeutic effects on the central nervous system (CNS). Studies have shown that EMF can activate voltage‐gated sodium channels (VGSCs) in different types of neurons, which increase intracellular calcium levels necessary for neural differentiation, survival, and regeneration.^[^ ^50^, ^51^ ^]^ Recently, researchers have focused on electromagnetized gold nanoparticles (GNPs) as a potential treatment for neurodegenerative disorders, such as Parkinson's disease, and to promote neurogenesis.^[^ ^52^, ^53^, ^54^ ^]^ Moreover, the use of GNPs in combination with EMF has shown promising results in both in vitro and in vivo studies, making it a potential therapy for CNS disorders including TBI.^[^ ^55^ ^]^

**Scheme 1:**
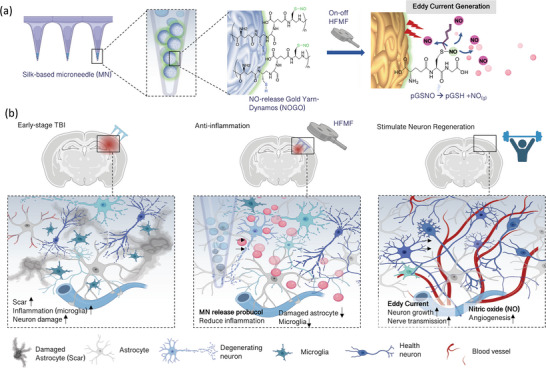
The high‐frequency magnetic field (HFMF)‐responsive NO‐release gold yarn‐dynamos (mNOGO) is a novel device that enables selective activation of traumatic brain injury (TBI) treatments. (a) The schematic illustrates the mNOGO's construction, which involves conjugating the NO donor S‐nitrosoglutathione (GSNO) with gold yarn (GY). Upon irradiation with HFMF, mNOGO releases NO and triggers electrical currents. (b) Loading the mNOGO into a silk microneedle (MN) facilitates targeted delivery for on‐demand NO and electron delivery during various stages of TBI treatments, leading to angiogenesis, neurogenesis, and functional recovery.

Here, inspired by neuron repair promoted by the physiology of NO and an electric stimulus, we propose high‐frequency magnetic field (HFMF)‐responsive NO‐release gold yarn‐dynamos (mNOGO) featuring triggered NO release and an electric stimulus for the selective activation of traumatic brain injury (TBI) treatments. Comprising a conjugated NO donor (poly(S‐nitrosoglutathione), pGSNO) with gold yarn‐dynamos (GY), the assembled mNOGO exhibits HFMF‐induced currents and burst release of NO (**Scheme** **1**a). After embedding mNOGO in a silk microneedle (MN), the implantable device in TBI delivered on‐demand NO release and electrons (Scheme 1b). At the early stage of TBI, it reduces the severe the infiltration of microglia and activated astrocytes, mitigating the formation of glial scaring. Later, the synergistic effects of currents and sustained release of NO induce angiogenesis, neurogenesis, and functional recovery in vivo.

## 2 Results and Discussion

### 2.1 Synthesis and Characterization of GY and mNOGO

**Figure 1:**
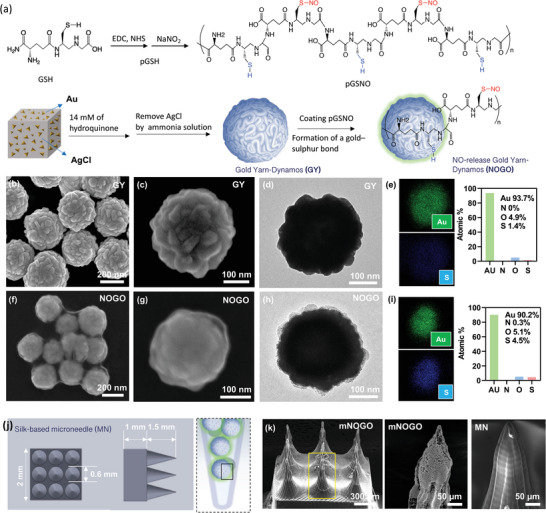
Preparation and characterization of gold yarn‐dynamos (GY) and mNOGO. (a) Schematic illustration of synthesis of mNOGO. SEM, TEM images and element mapping of(b)–(e) GY and (f)–(i) mNOGO. (j) A 3D printing design of the silk‐based microneedle. (k) SEM images of mNOGO and MN.

This versatile mNOGO was prepared using a GY‐assisted approach employing porous conductor nanoparticles comprising ligaments and conjugated with NO donor (poly(S‐nitrosoglutathione), pGSNO) in **Figure** **1**a. First, the synthesis of polyglutathione (pGSH) was performed by reaction of GSH, 1‐ethyl‐3‐(3‐dimethylaminopropyl)carbodiimide (EDC) and N‐hydroxysuccinimide (NHS), which commonly used as coupling agents to facilitate the reaction between a carboxylic acid and an amine to form an amide bond. Via the formation of an activated ester intermediate from the carboxylic acid in the presence of EDC, it reacts with the amine in the presence of NHS to form the amide.^[^ ^56^ ^]^ Then, pGSH and sodium nitrite were reacted in cold HCl solution (0.625 M) to form the pGSNO. The molecular weight of pGSNO was determined to be 31 kDa using MALDI‐coupled time‐of‐flight mass spectrometry (MALDI‐TOF MS). In FTIR spectra of pGSH, and pGSNO, S‐H bonding signal is decreased in pGSNO due to the replacement of S─H bonds by S─NO bonds (Figure S1, Supporting Information).

The GY were prepared by a seed‐growth method, where cubic silver chloride (AgCl) used as the sacrificing substrate, and the gold ligament was selectively deposited on the surface (Figure 1a). During the process, cubic AgCl was formed in advance by using a capping agent, namely, poly(vinylpyrrolidone) (PVP), and a silver nitrate ion‐exchange approach in ethylene glycol. Then, the gold seed was deposited in the defects of the cube's surface.^[^ ^57^ ^]^ By tuning a reducing agent (hydroquinone), porous GY with gold ligaments could be observed in few minutes. Finally, pGSNO was conjugated on GY through a process called a thiol‐gold reaction.^[^ ^58^ ^]^

The results from scanning and transmission electron microscopy (SEM and TEM), as shown in Figure 1b–d, indicate that GY particles with a mean diameter of ≈240 nm displayed roughness when the hydroquinone concentration was 14 mM. When the concentration of hydroquinone is reduced from 7 to 56 nM, it is to observe an increase in the size of particles as a result of gold particle growth (Figure S2, Supporting Information). The surface of GY exhibited distinct rough features and unique gold ligaments, providing increased surface area for potential chemical modification. Additionally, energy dispersive spectroscopy (EDS) mapping analysis demonstrated the presence of Au elements on the GY particles, as illustrated in Figure 1e. Following the conjugation of the NO donor pGSNO with GY, resulting in mNOGO particles, the surface of the particles became noticeably smoother, as shown in Figure 1f. Upon closer examination, the surface of GYs appeared to be covered by a semi‐transparent and polymer thin layer, indicating successful conjugation of pGSNO on GYs, as depicted in Figure 1g,h. The thickness of the pGSNO layer on GYs was estimated to be between 5 to 10 nm. Energy dispersive spectroscopy (EDS) analysis indicated a stronger presence of sulfur element compared to GY alone, further confirming the successful conjugation of pGSNO on GYs (Figure 1i).

Microelectrode arrays (MEA) are small devices shaped like microneedles that can be implanted directly into the brain. These arrays contain multiple small metallic probes and provide electrical stimulation with minimal invasion.^[^ ^59^, ^60^ ^]^ Inspired by the MEA design, mNOGO was loaded into silk‐based microneedles (MN) for in vivo implantation. To prepare the MN, a negative mold was created using polydimethylsiloxane (PDMS) and 3D‐printed MN, and the mold was then filled with methacrylated‐silk (silk‐MA) hydrogel mixed with mNOGO particles (Figure 1j). The mixture was centrifuged, cured, and then removed from the mold, resulting in a 3D‐printed MN that closely matched the original design (Figure S3, Supporting Information). The size and morphology of the resulting MN could be adjusted to fit various wound shapes by scanning and software, increasing the efficiency of treatment. Scanning electron microscopy (SEM) images of the mNOGO@MN surface showed that the tip was rougher than that of regular MNs, and many mNOGO particles were observed inside the MN (Figure 1k). These results suggested that mNOGO particles were densely loaded into the tips of the MN, which could potentially induce electrical stimulation to promote nerve regeneration upon an HFMF irradiation.

### 2.2 Current Production and NO Release upon HFMF Irradiation

**Figure 2:**
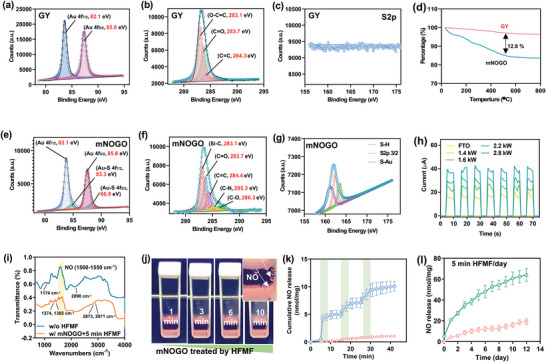
Current production and NO release from mNOGO. (a)–(c) XPS spectrum of GY. (d) TGA analysis of GY and mNOGO. (e)–(g) XPS spectrum of mNOGO. (h) *I–T* curve of them NOGO under the intermittent application of the HFMF. (i) IR spectrum of mNOGO with or without HFMF treatment. (j) The image of mNOGO treated by HFMF in water. (k) Cumulative NO release pattern of mNOGO under intermittent application of HFMF (2.8 kW and 1 MHz) (*n* = 5). The high‐frequency magnetic field (HFMF) was cyclically activated for 5 min (yellow area) and then deactivated for 5 min (white area), in an alternating pattern. (l) The NO release from mNOGO was measured after incubation in PBS at 37 °C, with or without daily 5 min HFMF treatment. Data represent the mean ± SD (*n* = 5).

High‐resolution X‐ray photoelectron spectrometry (HRXPS) is a powerful technique used to study the chemical bonding of materials. In this study, HRXPS was employed to investigate the bonding of GY and mNOGO. The analysis of GYs using the Au 4f, C1s, and S 2p signals revealed the presence of gold bonding and carbon bonds from PVP and hydroquinone, but no peak adsorption was observed for S2p (**Figure** **2**a–c). This observation suggests that no sulfur in the sample was chemically bonded to any of the elements. After conjugation of pGSNO, TGA analysis indicated the organic ratio of mNOGO, suggesting that pGSNO was successfully conjugated to GY (Figure 2d). HRXPS analysis also revealed the formation of an Au─S bond attributed to the thiol group of pGSNO and GY (Figure 2e–g). The binding energy of the bonds increased in the spectrum of Au 4f, indicating the formation of a stronger bond. Furthermore, the formation of an Au‐S covalent bond was detected at a binding energy of 162.9 eV, which further confirms the formation of an Au─S bond. These results demonstrate that pGSNO was successfully conjugated to GY, forming an Au─S bond.

Figure 2h shows the *I–T* curve of GY in an on‐off high‐frequency magnetic field (HFMF) with a strength of 2.8 kW and a frequency of 1 MHz, which was switched every 5 s. The results indicate that GY can generate eddy currents that can be manipulated by the strength of the HFMF. Eddy currents are generated when a conductor, such as GY, is exposed to a changing magnetic field. When the magnetic field changes, it induces a voltage in the conductor, which generates a current. These currents circulate within the conductor in closed loops, perpendicular to the direction of the magnetic field, and are known as eddy currents. The strength of the HFMF determines the amplitude of the eddy currents generated in the GY material. The on‐off switching of the magnetic field every 5 s creates a pulsing effect that causes the eddy currents to increase and decrease in amplitude accordingly.

The loading of the NO donor pGSNO in NOGO via the formation of an Au‐S bond allows for the cleavage of the S─NO bond, resulting in the release of NO. To investigate the cleavage of the bond under high‐frequency magnetic field (HFMF) treatment, infrared spectroscopy (IR) analysis was performed. As shown in Figure 2i, the cleavage of the S─NO bond was detected at 764 cm^−1^ (─S─N═) and 1505 cm^−1^ (─N═O) by Fourier‐transform infrared spectroscopy (FTIR) after HFMF treatment. It also reveals a strong peak result at 2090 cm^−1^, indicating that the absorption of the C─N band of GSH is stretched. The characteristic doublet at 1374 and 1385 cm^−1^ was assigned to the deformation vibration of the isopropyl group in GSH. In addition, GSH exhibits different peaks at different functional groups, among which the most obvious peaks are 2971 cm^−1^ for C─H asymmetric stretching, 2873 cm^−1^ for C─H symmetric stretching, and amide of 1634 cm^−1^. The C═O of this group is the bending vibration of CH_2_− and ‐CH_3_ respectively. In addition, there is an absorption peak at 1174 cm^−1^, which is attributed to the ν(C = O) of the catechol moiety. The results indicated that the HFMF treatment causes a pulsing effect that can induce the generation of eddy currents in the material, which can lead to the cleavage of the S─NO bond. The bubbles observed in the solution with increasing time of HFMF treatment suggest that NO gas is released due to the cleavage of the S─NO bond (Figure 2j). The released bubbles are collected and analyzed by gas chromatography (GC) in Figure S4 (Supporting Information).

The Griess assay, a common method for detecting NO, showed a gradual release of NO under stimulation. By adjusting the duration of HFMF application, the on‐demand release of NO could be regulated (Figure 2k). When mNOGO was repeatedly exposed to HFMF (2.8 kW, 1 MHz), burst releases of NO were synchronized with the stimulus. During HFMF‐on states, 4.2 nmol mg^−1^ of NO was released, while during HFMF‐off states, a subtle release of 0.22 nmol mg^−1^ of NO was detected. After 40 min of HFMF stimulus, the release of NO reached 9.8 nmol mg^−1^, and the NO release patterns exhibited a linear relationship to treatment time. Furthermore, DAF‐FM (4‐Amino‐5‐Methylamino‐2′,7′‐Difluorofluorescein) is a fluorescent probe commonly used to measure nitric oxide (NO) levels. Nitric oxide (NO) levels were also assessed using DAF‐FM, with changes in fluorescence intensity serving as an indicator. The release patterns, depicted in Figure S5a (Supporting Information), closely resembled those detected by the Griess assay. Figure 2l shows that applying HFMF to mNOGO for 5 min everyday results in continued and steady NO release, which is similar to the slow NO release observed from mNOGO incubated in PBS at 37 °C without HFMF. The mechanism of NO release from mNOGO triggered by continuous and pulsatile application of HFMF is believed to be the electric decomposition of GSNO by the auto‐catalytic action of thiyl radicals.^[^ ^61^ ^]^

The exact mechanism of eddy currents inducing the release of nitric oxide is still not clear. An electric field typically does not directly break chemical bonds. However, in certain cases, an electric field can indirectly affect chemical bonds. For example, when applied electric field, it decreases the dissociation energy of weak bond and stabilizes the product relative to the reactant due to their different dipole moments.^[^ ^62^ ^]^ When an electric field is applied to a chemical system containing weak bonds like thiol (S─H) bonds, it can alter the distribution of electron density in the bond.^[^ ^63^, ^64^ ^]^ In the context of thiyl radicals, an applied electric field aligned in a specific way could potentially reduce the dissociation energy of the thiol (S─H) bond, making it easier to break. This means that less energy would be required to initiate the homolytic cleavage of the thiol bond, leading to the formation of thiyl radicals (R‐S•). Additionally, the applied electric field may also influence the relative stability of the reactant (thiol) and the product (thiyl radical). Thiyl radicals contain an unpaired electron, which introduces a magnetic moment and makes them paramagnetic. On the other hand, the reactant (thiol) typically does not possess an unpaired electron and is diamagnetic.^[^ ^65^ ^]^ To assess the production of thiyl radicals upon exposure to magnetic fields, we employed Electron Paramagnetic Resonance (EPR) spectroscopy in conjunction with 5,5‐Dimethyl‐pyrroline N‐oxide (DMPO) spin traps on a Bruker Elexsys E580 EPR Spectrometer. The findings conclusively revealed the formation of thiyl radical adducts following treatment with HFMF (Figure S5b, Supporting Information).

The concentration of NO released upon 5 min of HFMF irradiation was lower than 400 nM, which is suitable for vasodilation, anti‐inflammation, and tissue repair. Furthermore, mNOGO@MN displayed similar NO release behaviors, as the diffusion of NO to the solution was not affected by the MN. Furthermore, the temperature of GYs or mNOGO solution upon an HFMF for 10 min was monitored. There was only ≈2–3 °C increase in temperature. The identical temperature of solution did not trigger NO release. Moreover, to simulate the local temperature of GYs or NOGO in vivo, an agarose gel was employed as a surrogate for brain tissue. This selection stemmed from existing literature, which identifies both brain tissue and agarose gel as poroelastic substances.^[^ ^66^, ^67^ ^]^ Then, the particles were injected in the gel. The temperature of GYs or NOGO in the vicinity upon a 10 min exposure to HFMF showed a negligible rise of ≈1 and 2 °C.

To understand the advantages of the GY structure, gold nanoparticles (GNPs) with an average diameter of 10 nm were synthesized and compared with GY. First, compared to GYs, GNP does not provide a cavity surface, resulting in aggregated and aggregation of pGSNO and GNP (Figure S6a,b, Supporting Information). Furthermore, due to the sizes, the induced currents of GNPs were lower than GYs (Figure S6c, Supporting Information). The reasons were attributed to the ligament structures of GYs. Since the induced eddy current decreases exponentially with the surface depth, the rough surface caused by the structure can induce an effective current called the skin effect under electromagnetic stimulation. For in vivo neural stimulation, the common current was ranged from few to hundred microamperes.^[^ ^68^ ^]^ Moreover, GY showed better on‐demand NO release by external HFMF compared to GNP. This may be due to the uniform pGSNO coating and efficient current induction (Figure S6d, Supporting Information).

### 2.3 In Vitro Study and the Differentiation of Neural Stem Cells (NSCs) by mNOGO

**Figure 3:**
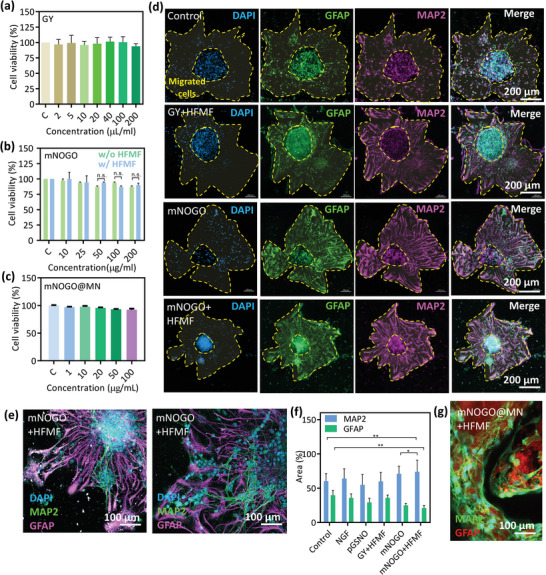
In vitro study and electromagnetized simulation‐induced neuron stem cell (NSC) differentiation. (a)–(c) Cell viability of NIH‐3T3 cells after incubation with GY, mNOGO+HFMF, and mNOGO@MN, respectively (*n* = 5, mean ± s.d., one‐way ANOVA with Tukey's multiple comparison test). (d) Confocal images of NSCs differentiated from embryonic cerebral cortical neurospheres after 7 days in culture, showing anti‐MAP2 (purple) and anti‐GFAP (green) immunoreactions of differentiated neurons and astrocytes, respectively. (e) Neural differentiation morphology of NSC sphere under mNOGO+HFMF.( f) Quantification of MAP2 and GFAP of migrated cell neurospheres in the migrated zone. Data represent mean ± SD (*n* = 4 per group). (g) Confocal images of NSCs treated with mNOGO@MN+HFMF.

To assess cytotoxicity, NIH‐3T3 cell lines derived from mouse embryonic fibroblasts were exposed to GY, mNOGO, and mNOGO@MN. **Figure** **3**a shows that the cell viability of NIH‐3T3 cells was more than 95% while incubating with GY, indicating no toxicity of GY to cells. Similarly, mNOGO also showed good biocompatibility with and without HFMF treatment (Figure 3b). When the cells were exposed to HFMF for 5 min, no significant toxicity was detected, suggesting that the NO and current did not have a significant impact on the cells. Additionally, the toxicity of the particles was further reduced when they were embedded in MN (Figure 3c). Furthermore, the GY and mNOGO‐loaded silk‐MA hydrogels were co‐cultured with NIH‐3T3 for 2 days. According to the images presented in Figure S7 (Supporting Information), it is evident that cell adhesion on the gel substrate is observed, suggesting a favorable cell affinity.

To validate the ability of mNOGO to release nitric oxide (NO) upon HFMF stimulation in cells, we utilized the DAF‐FM reagent for testing. DAF‐FM DA is a fluorescent probe that can permeate cells and detect intracellular NO levels. DAF‐FM reacts with NO+ equivalents, such as nitric anhydride (N_2_O_3_), which are produced through the autoxidation of NO. Figure S8 (Supporting Information) illustrates the results, demonstrating that mNOGO can release NO when subjected to HFMF stimulation. As a result, the cells exposed to mNOGO exhibit intense green fluorescence, indicating a robust NO production.

The effects of mNOGO on the differentiation of neural stem cells (NSCs) were evaluated with and without HFMF treatment. Primary NSCs were extracted from embryonic cerebral cortical neurospheres (Figure S9, Supporting Information). NSCs were divided into five groups: 1) control group, 2) GY+HFMF, 3) mNOGO, 4) mNOGO+HFMF. For HFMF‐treated groups, HFMF at a power of 2.8 kW and frequency of 1 MHz was applied for 5 mins per day until the NSCs were fixed. At day 7, NSCs demonstrated differentiation (Figure 3d). The expression of a neuron marker (MAP‐2) and glial fibrillary acid protein (GFAP, astrocyte marker) was analyzed to assess differentiation fate. Compared to the control group, the NSCs treated with GY+HFMF and mNOGO+HFMF exhibited obvious sprouting, indicating current‐induced neural‐related cell differentiation of NSCs. With exposure to HFMF, the NSCs exhibited efficient neuron differentiation with significant increases in the number of MAP‐2‐positive neurons.

Electrical stimulation promotes neuronal sprouting by activating intracellular signaling pathways and enhancing the self‐renewal capacity of neuronal connections between two neurospheres (Figure 3e), as it affects cellular responses and triggers specific molecular mechanisms as follows.^[^ ^69^, ^70^, ^71^ ^]^ 1) Calcium signaling pathway: electric stimulus can lead to the influx of calcium ions into neurons through voltage‐gated calcium channels. Increased intracellular calcium levels play a critical role in triggering various cellular processes, such as neurotransmitter release. 2) cAMP (cyclic adenosine monophosphate) signaling pathway: electric stimulation can activate adenylate cyclase, an enzyme responsible for producing cAMP. Elevated cAMP levels can initiate a cascade of events that affect neuronal activity and synaptic plasticity. 3) MAPK (mitogen‐activated protein kinase) signaling pathway: electric stimulus can activate MAPK pathways, including ERK (Extracellular Signal‐Regulated Kinase), JNK (c‐Jun N‐terminal Kinase), and p38 MAPK. These pathways are involved in regulating cell differentiation. 4) Wnt signaling pathway: electric stimulus can influence the Wnt pathway, which is essential for neural development and adult neurogenesis. Wnt signaling is involved in promoting neuronal differentiation and synaptic plasticity. 5) BDNF (brain‐derived neurotrophic factor) signaling pathway: electric stimulus can induce the release of BDNF, a neurotrophin that supports neuronal survival, growth, and synaptic plasticity. When neurons are subjected to an electric stimulus, it causes the activation of specific signaling pathways within the cells. These pathways are responsible for transmitting information and coordinating various cellular responses, facilitating neuronal connections between the two neurospheres.

The quantification of sprouting area treated by mNOGO+HFMF showed great neuron differentiation because both GSNO and electrons were beneficial to neuron differentiation (Figure 3f). The mNOGO‐treated NSCs preferred to differentiate into astrocytes rather than neurons. Physical and chemical stimulations to NSCs resulted in enhanced neuron differentiation since the electric signal and NO participate in the circulation of cells as well as eliminate oxidized substances to achieve neuroprotection, thus assisting the elongation of neurons.^[^ ^72^ ^]^ Furthermore, mNOGO loaded in MN also displayed long and dense neurons, and its morphology was similar to the particles alone group (Figure 3g). The quantification of neuron and astrocyte differentiation was also given in Figure S10, (Supporting Information).

Induction of inducible nitric oxide synthase (iNOS) and other nitric oxide synthase (NOS) isoforms in response to external agents can result in elevated nitric oxide (NO) levels within cells, influenced by the concentration of NO in the surrounding microenvironment and other reactive nitrogen species (RNS) derived from NO. The presence of high intracellular NO concentrations is typically associated with NO‐mediated toxicity, while lower levels of intracellular NO provide more opportunities for NO‐mediated cellular signaling. In Figure S11, (Supporting Information), the results of the Live/Dead experiment on neural stem cells (NSCs) are presented. The fluorescent indicator calcein‐AM, which indicates live cells, showed a close correlation with RNS‐dependent induction, meaning that higher cytotoxicity corresponded to greater RNS induction. Both GY and mNOGO were used at a concentration of 20 µg mL^−1^ in this experiment. The findings suggest that NSCs did not undergo apoptotic behavior, regardless of whether they were exposed to HFMF stimulation or not.

Excessive production of nitric oxide (NO) triggered by inflammatory signals is considered a key factor in the development of several neurodegenerative disorders. To assess the neuroprotective effects of different substances against glutamate‐induced excitotoxicity, various particles were applied to neural stem cells. When neurons are damaged by glutamate, the structure of astrocytes becomes fragmented, and their fluorescence intensity diminishes (Figure S12, Supporting Information). However, treatment with silk fibroin and mNOGO partially restored the GFAP structure, a marker of astrocytes. Notably, the mNOGO+HFMF treatment group demonstrated neuronal protection, with GFAP morphology resembling that of the control group. These findings indicate that the mNOGO+HFMF treatment have neuroprotective effects.

### 2.4 In Vivo Study of mNOGO on TBI

**Figure 4:**
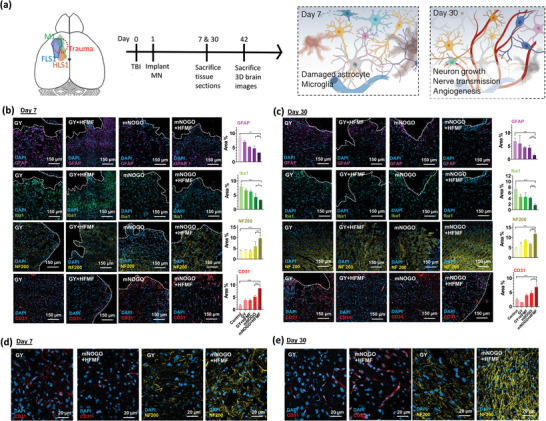
Animal study of traumatic brain injury (TBI) and treatment schedules. (a) Left panel: illustration of TBI and experimental timeline for treatment and sacrifice/analysis. Right panel: short‐ and long‐term stages of repair. (b),(c) Confocal laser scanning microscopy (CLSM) images of areas adjacent to the injury site, showing astrocytes (purple, stained with GFAP), microglia/macrophages (green, stained with Iba1), neurofilament cells (yellow, stained with NF200), and blood vessels (red, stained with CD31) at 7 and 30 days post‐injury, respectively. Blue fluorescence represents nuclei stained with DAPI (*n* = 5, mean ± s.d., *p < 0.05, **p < 0.01, one‐way ANOVA with Tukey's multiple comparison test). (d),(e) High‐magnification CLSM images of blood vessels and neurofilament cells at 7 and 30 days post‐injury, respectively.

To investigate the potential of stimulating neurons in the microenvironment for brain tissue repair, female C57BL/6 mice (7 weeks old) were used. To induce traumatic brain injury (TBI), a flat‐end puncher (2 mm in diameter) was used to remove brain tissue at the motor cortex to a depth of 1.5 mm (**Figure** **4**a). The materials were implanted one day after TBI, followed by the application of HFMF until sacrifice on day 7, 30, and 42. Immunohistochemistry staining was performed on harvested brains to evaluate the cellular response in the injury area. Mice sacrificed on day 7 were used to observe short‐term changes in the wound and were divided into five groups: 1) untreated group, 2) GY, 3) GY+HFMF, 4) mNOGO@MN, and 5) mNOGO@MN+HFMF. In TBI, a robust immune response involving a variety of immune cells contributes to rapid metabolism.^[^ ^73^ ^]^ In this case, we employ microelectrode arrays for implantation and neural repair. To assess degradation, we introduced GY‐NOGO nanoparticles and mNORO into mice with TBI. The results showed that 90% of the GY‐NOGO nanoparticles were metabolized within 7 days. In contrast, mNOGO remained in the brain longer, lasting more than 14 days (Figure S13a, Supporting Information). To ensure long‐term treatment, all particle treatment groups loaded the particles into MN (Figure S13b, Supporting Information).

The morphology of astrocytes was analyzed using GFAP (glial fibrillary acidic protein). In comparison to the untreated group, the groups treated with GY, mNOGO, and/or HFMF showed fewer and relatively loose astrocytes, as demonstrated in Figure 4b (CLSM images of untreated groups were provided in Figure S14a, Supporting Information). The quantification of images was conducted by randomly selecting 3 slices per animal (*n* = 4) and 4 regions of interest (ROIs) in the traumatic regions. As illustrated in Figure 4b, the reduced expression of GFAP after various treatments could be attributed to the microporosity of the hydrogel. The decreased presence of astrocytes in the trauma area resulted in tissue recovery benefits. The quantifications of fluorescence intensities for in vivo immunohistochemistry analysis were also added in Figure S14b (Supporting Information). Quantification of fluorescence intensity and area showed good correlation.

The immune response of traumatic injury and treatments targeting microglia cells were assessed by using ionized calcium‐binding adaptor molecule‐1 (Iba‐1). Both the untreated and material groups showed a significant number of microglia, as depicted in Figure 4b. In contrast, the groups treated with mNOGO exhibited a much lower amount of the Iba1 marker, indicating that applying mNOGO can substantially reduce the immune response. This reduction is achieved through the release of NO, which lowers the ROS level of the microenvironment. Furthermore, the group treated with mNOGO without HFMF had ≈1.2% more microglia than the groups treated with HFMF. Thus, treatment can significantly reduce the immune response by applying MN to decrease apoptosis and drugs to minimize the harmful factors of the microenvironment. Additionally, regenerated neurofilament cells were observed using the axonal marker Neurofilament 200 (NF200). The GY+HFMF and mNOGO+HFMF groups showed a stronger NF200 marker than other groups (Figure 4b). The application of a magnetic field induced GY to generate electric current, which promoted nerve cell growth. The quantifications of fluorescence intensities were given in Figure S14b (Supporting Information).

Figure 4c shows that after 30 days, the astrocytes in all groups were more apparent and denser than after only 7 days. The astrocytes in the untreated group were dense and had become scars. In contrast, the groups treated with MN had fewer and looser astrocytes (CLSM images of untreated groups were provided in Figure S14a, Supporting Information). GFAP area quantification of these groups was ≈4% lower than that of the untreated group. Moreover, the apoptosis of original cells along with the lack of newborn cells resulted in a larger cavity at the injury site. The Iba1 area quantification of the untreated group was ≈5.3% higher than that of the mNOGO+HFMF group, indicating that the release of pGNSO by MN could reduce immune response in the microenvironment. Additionally, the application of a magnetic field induced electric stimulation in the GY+HFMF and mNOGO+HFMF groups, promoting the expression of neural cells in these two groups more than in other groups.

TBI often results in cell apoptosis and blood vessel necrosis at the site of injury. The morphology of blood vessels can serve as an indicator of the level of recovery around the injury site. In the animal study, the blood vessels were stained with the CD31 marker at 30 days post‐surgery. In Figure 4c, clear vascular formation was observed in the treated groups, and some vessels longer than 80 µm were detected in the mNOGO+HFMF group. This result indicates that improved cell migration and vascular‐like network formation guided by pGSNO and HFMF leads to axonogenesis. However, only a few CD31 signals were observed in the trauma cavity in the control group. The CD31 area quantification of the mNOGO+HFMF group was ≈3.5% higher than that of the untreated groups, indicating that GSNO can release NO in the microenvironment and activate growth factors to stimulate angiogenesis. Thus, this group can recover blood vessels and tissue well. Figure 4d,e show enlarged CLSM images for the blood vessels and neurofilaments, indicating improvements by the mNOGO treatment.

NO is of low reactivity but produces detrimental RNS such as peroxynitrite (ONOO^−^) in the diffusion‐controlled reaction between NO itself and O_2_^−•^ (superoxide anion).^[^ ^74^ ^]^ To assess RNS effects on inflammatory and immune responses of in vivo, the brains with were collected on day 7 post‐treatment for immunological evaluation. The levels of immune factors, including tumor necrosis factor‐α (TNF‐α), IL‐1β and IL‐6 in lung tissues treated by particles were detected by ELISA kits. The levels of TNF‐α, IL‐1β and IL‐6in the groups treated with silk fibroin, mNOGO and mNOGO+HFMF were slightly decreased compared with the levels in the control group, suggesting the RNS effects in brain. Furthermore, the axonal morphology around the injected area did not show significant damage, suggesting that the treatment with minor RNS did not harm the tissue and neurons (Figure S15, Supporting Information). Moreover, liver and kidney function were evaluated 72 h post‐implantation following different treatments. Notably, no substantial alterations were observed in the functions of the liver and kidneys, underscoring the compatibility of the materials used (Figure S16, Supporting Information).

### 2.5 Whole‐Brain Imaging after mNOGO Treatment

**Figure 5:**
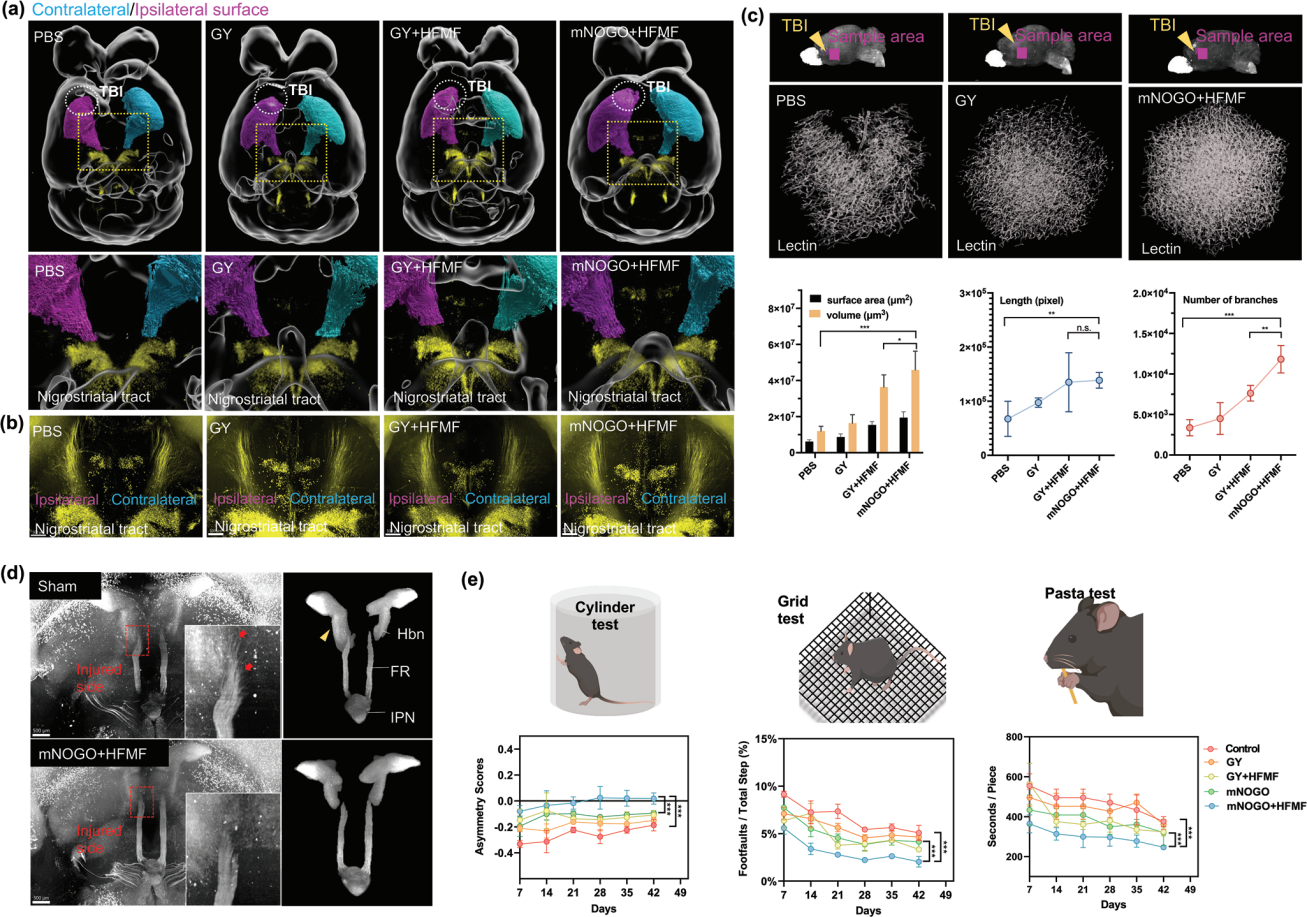
Whole‐brain imaging analyses and animal behavior tests of different treatments for traumatic brain injury (TBI) in mice. (a) Reconstructed 3D images of the whole brains of mice with TBI treated with various materials, with dopamine neurotransmission and tyrosine hydroxylase (TH) displayed in color. The injured side (right brain) is colored purple, and the normal side (left side) is colored blue. (b) Enlarged images of the brain to show the morphologies of TH. (c) Brain images of lectin for vascular imaging at peri‐trauma regions, with quantification of blood vessel length, surface area, and vessel branches (*n* = 5, mean ± s.d., *p < 0.05, **p < 0.01, one‐way ANOVA with Tukey's multiple comparison test). (d) Brain images stained by Choline acetyltransferase (ChAT) that provides insight into the function of cholinergic neurons in different parts of the nervous system. (e) Behavior tests (cylinder, grid, and pasta tests) after treatment with various conditions (*n* = 5, mean ± s.d., *p < 0.05, **p < 0.01, one‐way ANOVA with Tukey's multiple comparison test).

TBI can disrupt the normal functioning of dopamine neurotransmission in the brain. To assess dopamine neurotransmission in the brain, tyrosine hydroxylase (TH), a key enzyme in the synthesis of dopamine in dopaminergic neurons, was tracked to observe dopamine in the striatum in the whole brain (**Figure** **5**a and Movie S1, Supporting Information).^[^ ^75^, ^76^ ^]^ At 42 days post‐TBI, the left brain (blue) performed better than the right brain (purple) in all groups, as shown in the 3D images in Figure 5a. Notably, the untreated group's injured side had a significant groove, indicating difficulties in natural brain repair. In contrast, the mNOGO+HFMF group exhibited greater symmetry between the left and right brains, with better signal transmission in the brain than other groups, indicating gradual repair of the injured side (Figure 5b). Closer examination revealed that the density of TH expression at the injured site was higher in the mNOGO+HFMF group than in other groups, likely due to tissue regeneration.

The formation of blood vessels, or angiogenesis, is a critical factor in regulating tissue regeneration following injury. In this study, vessel density was evaluated using a 3D whole‐mouse‐brain clearing technique, and blood vessels were labeled with Lectin, a commonly used tool for visualizing blood vessels that binds to glycoproteins in the glycocalyx and basal membrane of endothelial cells. As illustrated in Figure 5c, blood vessels in mice with TBI treated with PBS and mNOGO+HFMF exhibited strong signals with characteristic vascular morphologies, resembling branch tube‐like structures (Movie S2, Supporting Information). Compared to the PBS group, the mNOGO+HFMF‐treated group displayed a higher density of vessels around the damaged brain (Figure 5c). This observation is consistent with the notion that NO levels below 400 nM can activate vascular endothelial growth. Other vascular images obtained from groups treated with alternative approaches demonstrated lower densities (Figure S17, Supporting Information). Following calculations, it was determined that the mNOGO+HFMF treatment resulted in a ≈210% improvement in angiogenesis in vivo at peri‐trauma regions.

TBI has been shown to cause a loss of cholinergic neurons in the medial septal nucleus,^[^ ^74^ ^]^ leading to a decrease in acetylcholine, an important neurotransmitter involved in learning and memory processes. The degradation of cholinergic neurons after TBI is thought to contribute significantly to cognitive deficits.^[^ ^77^ ^]^ The Habenulo‐Interpeduncular pathway (Hbn‐IPN pathway) is a highly conserved cholinergic pathway that has been identified as crucial to addiction, anxiety, and mood regulation,^[^ ^78^ ^]^ and also plays a crucial role in various behavioral domains, particularly inhibitory control and cognition‐dependent executive functions.^[^ ^79^ ^]^ To demonstrate cholinergic neuronal recovery, we compared the morphological changes of Hbn‐IPN complex and calculated the volumetric changes of fasciculus retroflexus (FR), the efferent fiber of Hbn, by employing whole mouse brain immunostaining with ChAT (Figures S18,S19 and Movie S3, Supporting Information). When calculating the volume changes, we normalized the volume of the injured side FR to that of the non‐injured side FR in order to avoid the influence of individual differences. In the control mouse, we observed an enlarged habenula (Figure 5d, yellow arrowhead) volume and diffused FR fiber terminals (red arrows), suggesting malfunction of the Hbn‐IPN complex.^[^ ^80^ ^]^ We also observed decreased FR volume and IPN intensity in the control mouse (Figure 5d), suggesting loss of cholinergic neurons. In contrast, the mNOGO+HFMF mouse exhibited a symmetrical Hbn‐IPN complex, and the relative volume of FR increased from 77% to 98% (Figure S20, Supporting Information). This observation suggests that the dropping FR volume and abnormal enlargement of Hbn were recovered in the mNOGO+HFMF, indicating a potential recovery of cholinergic neurons.

### 2.6 Animal Behavior Tests

To assess the efficacy of treatment in promoting recovery and restoring brain function, animal behavior tests were conducted following TBI and treatment. Female C57BL/6 mice at 7 weeks of age were divided into four groups (*n* = 5): 1) untreated, 2) GY, 3) GY+HFMF, 4) mNOGO, and 5) mNOGO+HFMF. Over a period of 42 days, animal behavior experiments were performed once a week to examine the long‐term effects of treatment. The cylinder test was used to evaluate forelimb movement (Figure 5e). As TBI was applied to the left brain, mice typically tend to use the left limb to explore the environment, resulting in decreased use of the right limb (positive) and increased use of the left limb (negative). The results of the cylinder test indicated that the untreated group performed progressively worse, while the frequency of forelimb use in the mNOGO‐treated groups tended to approach that of both limbs. To further assess the movement of other limbs, the grid test was employed (Figure 5e). In this experiment, foot‐faults of the hind‐limb were calculated. The untreated group exhibited the most foot‐faults in the grid test, while the mNOGO+HFMF group displayed fewer foot‐faults than the other groups. The uncooked pasta test (Figure 5e) was used to evaluate finger dexterity, with untreated mice taking a relatively long time to finish the pasta. The mNOGO+HFMF group exhibited the shortest time to finish the pasta on day 42, suggesting that this group had better finger dexterity compared to the other experimental groups.

## 3 Conclusion

In summary, this study presents a novel approach for repairing traumatic brain injury using an implantable microneedle containing GY and NO donors. The silk‐based microneedles, fabricated by 3D printed mold, effectively lower inflammation by displaying different phases. The conductive GY in the microneedles induced eddy currents under HFMF to promote the differentiation of neuron stem cells, while NO donors released NO to enhance the growth of neural stem cells. In vivo experiments demonstrated that the GY and NO donors‐loaded microneedles lowered astrocytes and immune responses, leading to improved nerve regeneration. Additionally, the microneedles facilitated angiogenesis with the assistance of HFMF, promoting the regeneration of neurons in vivo. The integration of these functions also improved limb symmetry and finger dexterity in animal behavior tests. Overall, these conductive materials have the potential for use in magnetoelectric‐mediated nerve therapy and in clinical settings, offering a promising avenue for the treatment of traumatic brain injury.

## Conflict of Interest

The authors declare no conflict of interest.

## Supporting information

Supporting Information

Supplemental Movie 1

Supplemental Movie 2

Supplemental Movie 3

## Data Availability

The data that support the findings of this study are available from the corresponding author upon reasonable request.
