# Supplementary material for: In Situ Forming of Nitric Oxide and Electric Stimulus for Nerve Therapy by Wireless Chargeable Gold Yarn‐Dynamos
Source: Adv Sci (Weinh). 2023 Oct 22;10(33):2303566. doi: 10.1002/advs.202303566 (PMC10667856; doi:10.1002/advs.202303566)
Supplement: Supplementary file 1 — Supporting Information [file ADVS-10-2303566-s004.pdf]

ADVANCED SCIENCE
Open Access

Supporting Information

for *Adv. Sci.*, DOI 10.1002/advs.202303566

In Situ Forming of Nitric Oxide and Electric Stimulus for Nerve Therapy by Wireless Chargeable Gold Yarn-Dynamos

Min-Ren Chiang, Ya-Hui Lin, Wei-Jie Zhao, Hsiu-Ching Liu, Ru-Siou Hsu, Tsu-Chin Chou, Tsai-Te Lu, I-Chi Lee, Lun-De Liao, Shih-Hwa Chiou, Li-An Chu and Shang-Hsiu Hu**

Supporting Information

In Situ Forming of Nitric Oxide and Electric Stimulus for Nerve Therapy by Wireless Chargeable Gold Yarn-Dynamos

Ming-Ren Chiang[a], Ya-Hui Lin[a,b], Wei-Jie Zhao[a], Hsiu-Ching Liu[a], Tsu-Chin Chou[c], Tsai-Te Lu[d-f], I-Chi Lee[a], Ru-Siou Hsu[g], Lun-De Liao[h], Shih-Hwa Chiou[i,j], Li-An Chu[a,b], Shang-Hsiu Hu[a]

[a]Department of Biomedical Engineering and Environmental Sciences, [b]Brain Research Center, [c]Institute of Analytical and Environmental Sciences and [d]Institute of Biomedical Engineering, [e]Department of Chemistry, National Tsing Hua University, Hsinchu 300044, Taiwan

[f] Department of Chemistry, Chung Yuan Christian University, Taoyuan, 320314, Taiwan

[g]Department of Chemistry, Stanford University, Stanford, CA 94305, U.S.A

[h]Institute of Biomedical Engineering and Nanomedicine, National Health Research Institutes, Miaoli County 35053, Taiwan

[i]Institute of Pharmacology, College of Medicine, National Yang Ming Chiao Tung University and, [j]Department of Medical Research, Taipei Veterans General Hospital, Taipei, 112304, Taiwan

E-mail addresses: shhu@mx.nthu.edu.tw; lachu@mx.nthu.edu.tw

Experimental Section

Synthesis of gold yarnballs (GYs)

The gold yarnballs (GYs) were synthesized using a seed-growth approach, employing cubic silver chloride (AgCl) as sacrificial templates onto which gold ligaments were selectively deposited.[1] The synthesis procedure is as follows: (1) Formation of cubic silver chloride (AgCl): A solution was prepared by combining 70 µl of $AgNO_3$ (100 mM) with 110 µl of $HAuCl_4$ (40 mM). (2) Addition of PVP solution: Subsequently, 4.5 ml of 100 mM PVP solution was added to the mixture while gently stirring at room temperature. The stirring was continued for 10 minutes. (3) Introduction of the reduction agent: The

reduction agent, 160 μl of 28 mM hydroquinone, was slowly added dropwise to the mixture at room temperature in the absence of light. As the reduction reaction proceeded, the solution gradually changed its color to light black, indicating the formation of gold yarnballs. The reaction was allowed to continue for 3 hours. (4) Removal of AgCl cores: To eliminate the AgCl sacrificial templates, 33% NH4OH solution was applied through repeated washing cycles performed three times. Excess water was subsequently used to wash and purify the resulting particles.

Synthesis of poly S-nitrosoglutathione (pGSNO) and mNOGO

To prepare the red colloidal product derived from glutathione, the following steps were performed. Briefly, a quantity of 1.53 of glutathione was dissolved in a hydrochloric acid (pH 4) solution.[2] Then, 0.095 g of 1-ethyl-3-(3-dimethylaminopropyl)carbodiimide (EDC) was added to the glutathione solution in acidic (pH 4.5) conditions. The reaction mixture was allowed to react overnight. After the completion of the reaction, the mixture was subjected to freeze-drying. The resulting product was then dissolved in 8 mL of ultrapure water (UPW) containing 2.5 mL of 2 M HCl, with an ice bath used for cooling. Then, Sodium nitrite ($NaNO_2$, 0.1725 g) was gradually added to the solution while maintaining a low temperature near 0°C. The mixture was stirred for 40 minutes. Following the stirring period, 10 mL of acetone was added to the red solution and further stirred for 10 minutes. Finally, the resulting red colloidal product was filtered, and the filtrate was washed three times, sequentially, with 1 mL of ice-cold water, 10 mL of acetone, and 10 mL of ether to remove impurities. The magnetic field (HFMF)-responsive NO-release gold yarnball (mNOGO) was prepared by adding 10 μg of pGSNO to 30 μg GY. The gold and thiol reaction was carried out at room temperature for 1 h. Then, remove excess pGSNO with deionized water.

Fabrication of Microneedles Using a Digital Light Processing (DLP) 3D Printer

In this experiment, microneedles were fabricated using a digital Llight processing (DLP) 3D printer (MiiCraft plus, Taiwan) operated by a computer connected via an RJ-45 cable. The printer was equipped with a light-emitting diode (LED) projector emitting light at a wavelength of 405 nm and a power of 12.5 mW. The 3D printed resin used for

microneedle fabrication consisted of a mixture of light-curable materials. The formulation included 17.8 g of 4-hydroxybutyl acrylate (4-HBA), 2 g of urethane-polyethylene glycol-polypropylene glycol (PU-EO-PO) monomer, and 0.2 g of Irgacure 819 as the initiator.[3] These components were thoroughly mixed and stored at room temperature in a dark environment. The design of the microneedle objects was created using software (SOLIDWORKS) prior to printing. The design files were then imported into a slicer software (MiiUtility 3.2) and uploaded to the 3D printer for printing. During the printing process, each layer had a thickness of 20 μm, and the curing time for each layer was set to 7 seconds. The printing stage was lowered to a position 100 μm above the resin pool after filling the printing pool with the resin. Layer by layer, the structure was polymerized and formed on the carrier (MiiCraft+).

After the printing process, the 3D resin microneedles were washed twice with 95% alcohol and deionized water to remove any residual uncured resin. Subsequently, they were subjected to further post-curing using an 18 W UVA lamp at room temperature for 30 minutes to enhance their mechanical properties and ensure complete curing. Finally, the 3D resin microneedles were dried in an oven at 60 °C overnight to remove any remaining moisture.

Fabrication of Silk-Methacrylic Anhydride (Silk-MA) Microneedles Loaded with mNOGO

First, silkworm cocoons were cut into small pieces and added to a Na_2CO_3 solution (0.05 M in 1 L) with 40 g of silkworm cocoons. The Na_2CO_3 solution with silkworm cocoons was boiled at 100 °C for 30 minutes to remove the sericin. The degummed silk was washed multiple times with distilled water. Second, the degummed silk was placed in a ventilated and dark place, such as a fume hood, to dry. The dried degummed silk was dissolved in 100 mL of 9.3 M lithium bromide (LiBr) at a ratio of 20 g of degummed silk, and the solution was heated at 60 °C for 1 hour until completely dissolved. Then, to the LiBr solution, 6 mL of methacrylic anhydride (MA) at 424 mM concentration was added. The solution was stirred at 300 rpm at 60 °C for 3 hours. The resulting solution was frozen at -20 °C and then dialyzed for 4 days. Finally, the solution was frozen overnight at -20 °C and lyophilized for 72 hours. The resulting powder, silk-MA, was stored at -20 °C.

Fabrication of Silk-MA Microneedles Loaded with mNOGO

The silk-MA solution consisted of 89 wt% water, 10 wt% silk-MA powder, 0.5 wt% N,N-methylenebisaceylamide (MBAA), and 0.5 wt% lithium phenyl (2,4,6-trimethylbenzoyl) phosphinate (LAP). MBAA acted as a cross-linking agent, forming a network structure to improve strength and elasticity. LAP acted as a crosslinking accelerator, speeding up the photocuring process. First, the silk-MA solution was added to a PDMS mold in an amount of 50 μL, along with 1 mg of NOGO particles. The solution in the mold was centrifuged at 4,900 rpm for 60 minutes. After centrifugation, the solution was irradiated with a point light source for 60 seconds to initiate photocuring. Second, a 2 μL solution of 10 wt% poly(vinyl alcohol) (PVA) (MW ~ 25,000 g/mol) was applied as a base and covered the photocured microneedles. The mold containing the microneedles was placed in a ventilated and dark place overnight to dry. Finally, the dry silk-MA microneedles were clamped out using tweezers for further use.

Characterizations of GYs

The average size and zeta potential of nanoparticles were analyzed by dynamic light scattering (DLS, Nano-ZS, Malvern). Samples dispersed into water in glass cuvette. The size distribution of nanoparticles was measured by the light hit particles in a period of time. Field-emission scanning electron microscope (FE-SEM, JSM-7000F, Japan), a transmission electron microscope (TEM, JEM-2100, Japan) were applied to observe the morphologies of nanoparticles. The elemental mapping of nanoparticles was performed by the energy dispersive spectroscopy (EDS) of TEM. For SEM analysis, all the samples were dried on the silicon wafers under room temperature, and gilded with an ultrathin platinum layer on the wafer to enhance the image quality taken in the experiments through the intensive electronic sputtering. For TEM analysis, nanoparticles were dried on the copper grid and took digital pictures of several locations on the grid to observe lattice of crystallite and obtain a representative set of images. High resolution X-ray photoelectron spectrometer (HRXPS, PHI Quantera SXM, Japan) can determine the surface composition of nanoparticles.

Griess Assay

The Griess reaction was applied to quantify the nitrite in aqueous media.[4] Briefly, the Griess reagent contained 40 mg of Griess powder and 1 mL of deionized water. To evaluate the sample of mNOGO, 100 mg of particles was added into 1 mL of deionized water. At different time points, 500 µL of the Griess reagent and 500 µL of the sample were mixed and waited for 15 min. The absorption of Griess reagent at wavelength of 540 nm was detected by UV-VIS Spectrometer (SP-8001, Metertech). The, the concentration of NO could be calculated by the calibration curve. For HFMF-treated groups, the solutions containing particles or pGSNO were subjected to HFMF for various times. Then, similar protocols were used to determine the nitrite in aqueous media. To assess the total amount of NO in mNOGO, mNOGO was heated to 70 ºC to cleave the S-NO bond, and NO release was monitored by Griess assay. After equilibrating for 24 h, the NO content was determined to be 100%. Cumulative release of NO (%) = release of NO/total amount of NO.

Electron Paramagnetic Resonance (EPR) spectroscopy

To assess the production of thiyl radicals upon exposure to magnetic fields, we employed Electron Paramagnetic Resonance (EPR) spectroscopy in conjunction with 5,5-Dimethyl-pyrroline N-oxide (DMPO) spin traps on a Bruker Elexsys E580 EPR Spectrometer. In a concise summary of the procedure, we prepared samples by incubating them in a nitrogen glove box in a phosphate buffer solution (200 mM) containing DMPO (100 mM). These samples were then loaded into a 100 ml capillary (Corning), sealed at both ends with Miniseal (Baxter). The capillary was subsequently positioned within a 4-mm quartz tube, which in turn was inserted into the ESR cavity. It's important to note that all sample preparations were conducted in a lightless environment, both with and without exposure to magnetic field treatments.

In Vitro Cytotoxicity of Materials

The cell toxicity of GY, NOGO and mNOGO was evaluated by PrestoBlue® cell viability assay. In brief, NIH-3T3 cells (derived from mouse embryonic fibroblasts) were cultured into 96-well tissue culture plate at a density of 1×10^4 cells per well in 100 µL of cell culture medium. At 24 h, the materials were co-cultured with the cells at 37 °C for 24

h, followed by extraction of the supernatant as the testing medium. On the other hand, the medium containing mNOGO can be testing medium directly without extraction. After incubation in fresh medium, the cells would be incubated in the previously prepared under different concentrations of extracted medium for another 24 h. After that, 10 μL of Presto Blue solution was added in each well and reacted for 10 min. ELISA reader (SynergyTM HT Multi-detection microplate reader, Bio Tek Instruments, Inc. USA) was used to read the absorbance value at excitation wavelength of 570 nm. The cell viability was determined by comparing with the unexposed cells.

In Vitro **Co-Culture of Cells with silk-MA and Microneedle**

For the observation, particle-loaded silk-MA films or MN were stained by red fluorescence dye (RITC, emission at 580 nm), and then, incubated in 2 mL medium with 1×10^4 cells/mL of NIH-3T3 cells in confocal dishes at 37 °C. After 24 h of incubation, 4',6-diamidino-2-phenylindole (DAPI) and Alexa Fluor 488 Phalloidin (F-actin) were used to stain the nucleus and cytoskeleton, respectively. After the staining, the excess chemicals were washed by PBS for three times and mounted with the Fluoromount™ aqueous mounting medium solution (Sigma-Aldrich). The samples were examined by a Carl Zeiss laser scanning confocal microscope (ZEISS LSM 800; Carl Zeiss, Oberkochen, Germany) which was equipped with a Plan-Neofluar (20×/0.5) and a Fluar objective (40×/1.30 and 63/1.40, oil immersion, Zeiss). The laser sources included 405, 488, 561 and 670 nm for DAPI (emission at 470 nm), GFP (emission at 510 nm), RITC (emission at 580 nm) and Cy5.5 (emission at 470 nm).

In Vitro **Differentiation of Neuron Stem Cells (NSCs)**

For NSCs culture, the confocal dishes were coated with poly-l-lysine (PLL) to assist NSCs with adhesion on the dish. First, the surface of confocal dish was modified by the oxygen plasma for 2 mins. Afterwards, the 1 mL of pH 7.4 tris buffer was added into the dish to wash it for 3 min for 3 times. After washed by tris buffer, 1 mL of PLL was added into the dish and waited for 30 min. Then, the dish was washed with tris buffer as the previous step for 3 times. Finally, the dish was covered by the 2 mL of tris buffer and placed in the laminar flow bench with UV light overnight. Before planting NSCs in the

dish, the dish was washed with deionized water for 3 times. The number of NSC spheres can be calculated by 24 well. Each dish was planted ~200 NSCs spheres. After NSCs spheres attaching on the dish stably (~8 h), the different materials were added to the dish to co-culture with NSCs. The medium to culture NSCs with differentiating was different from the maintaining medium. The differentiating medium was F12/DMEM containing with 1 % penicillin, 1 % N-2 supplement, and 0.2 % B-27 supplement. The NSCs were divided into five groups: (1) control group, (2) neuron growth factor-treated group (NGF), (3) pGSNO, (4) mNOGO, and (5) mNOGO+HFMF. According to groups requirements, 100 ng/mL of NGF, 10 μg/mL of mNOGO, and 16.8 μg/mL of pGSNO were dissolved in their medium and HFMF was applied for 5 mins per day. For HFMF-treated groups, HFMF at a power of 3.2 kW and frequency of 1 MHz was applied for 5 mins per day until the NSCs were fixed.

After 7 days, the NSCs were washed with PBS for three times and fixed by 70 % methanol solution for 5 min. The fixed NSCs were washed for 3 times again. Then, the samples were soaked in the blocking buffer containing 10 % BSA for 1 h at room temperature. After removing the blocking solution, the samples were then soaked in the primary antibodies: (1) GFAP: rabbit anti-glial fibrillary acidic protein (1:500 dilution). (2) MAP2: mouse anti-microtubule associated protein 2 (1:500 dilution). Afterwards, the NSCs were washed with PBS three times and stained by the secondary antibody for 2 h at room temperature. The secondary antibody: (1) Donkey anti-rabbit IgG (H&L) (1:500 dilution). (2) Goat anti-mouse IgG (H+L) antibody (FITC) (1:500 dilution). Subsequently, the samples were washed with PBS for three times and DAPI-mounting was applied to soak the samples. Laser scanning confocal microscope (ZEISS LSM 800; Carl Zeiss, Oberkochen, Germany) were used to observed. To calculate the percentage of differentiated cells of phenotype, the intensity of GFAP and MAP-2 positive cells were calculated of the area out of the neutrospheres to determine the astrocyte and neuron percentages in each field, respectively. This analysis was calculated by the image J software.

In Vivo Experiment

The surgical procedure accorded to the protocol approved by the Animal Care and Use Committee, National Tsing Hua University, Hsinchu, Taiwan. The silk-MA microneedles (MNs) were sterilized by ultraviolet light. C57BL / 6 mice (Female, 7 weeks) were divided into different groups: (1) untreated group, (2) GY, (3) GY+HFMF and (4) mNOGO+HFMF. The material in the MNs was added by an amount of 50 μL with 1 mg of particles. When applying traumatic brain injury (TBI) to mice, a hole on the skull was made by an electric drill. The site of TBI was on the left motor cortex (M1 & M2). Then, a 2 mm in diameter punch was adopted to give a 1.5 mm injury in depth. The MNs were implanted at one-day post-injury to avoid excessive swelling in the injured area. For AMF–treated groups, a magnetic field at a power of 2.8 kW and frequency of 1 MHz was applied for 5 mins per day until the mice were sacrificed.

Brain Collection and Immunofluorescence Staining

After sacrifice, brains of the mice were collected and fixed in 4% of paraformaldehyde (PFA) solution for 12 h, subsequently, soaked in 10% (10 mins), 15 % (10 min), 20% (30 min) and 30% (overnight) sucrose solution for dehydration. Afterwards, brains were embedded in optimal cutting temperature compound (OCT) and sectioned into slices under the thickness ~ 15 μm at 20 °C for immunofluorescence. The slices were fixed in methanol at -20 °C for 5 min and washed by PBS for three times. In order to remove non-specific binding of antibodies, the samples were immersed in the blocking buffer containing 5 % BSA, 0.1% of Tween20, and 0.25% of Triton X-100 in PBS for 1 h at room temperature. The samples were then soaked in following antibodies: (1) GFAP for astrocyte cells (rabbit, 1:200 dilution, Abcam), (2) NF200 for regenerated neurofilament (rabbit, 1:200 dilution, Abcam), (3) Iba1 for microglia cells (goat, 1:200 dilution, Abcam), (4) CD31 for vascular cells (rat, 1:200 dilution, Abcam) at 4 °C for 16 h. Afterwards, the slices were washed with PBS for three times and stained by the secondary antibody for 2 h at room temperature. The secondary antibody: (1) Donkey anti-rabbit IgG (H&L) 647 (1:200 dilution), (2) donkey anti-rabbit IgG (H&L) 647 (1:200 dilution), (3) rabbit anti-goat 488 antibody (1:200 dilution), (4) goat anti-rat 488 antibody (1:200 dilution). Subsequently, the samples were washed with PBS for three times and DAPI-mounting was applied to soak the samples. The morphology of all the stained sections was observed using a laser scanning confocal

microscope (ZEISS LSM 800; Carl Zeiss, Oberkochen, Germany). The intensity and the percentage of differentiated cells were analyzed by image J software.

3D Brain Analysis

To prepare the brains for analysis, the mice were first perfused with ice-cold PBS and then with SHIELD (Stabilization to Harsh conditions via Intramolecular Epoxide Linkages to prevent Degradation) perfusion solution.[5] The collected brains were soaked in the same perfusion solution at 4°C for two days. The tissues were then incubated in SHIELD-OFF solution at 4°C for two days and in SHIELD-ON solution at 37°C for another day. All reagents were prepared from SHIELD kits (LifeCanvas Technologies, South Korea) according to the manufacturer's instructions.

The SHIELD-processed brains were cleared using stochastic electro-transport (SmartClear Pro II, LifeCanvas Technologies, South Korea) with a constant current of 1.[6] A for around 5 days. The cleared brains were washed with PBST at room temperature for at least overnight. Immunolabeling was performed using the modified eFLASH2 method and active SmartLabel System (LifeCanvas Technologies, South Korea). To do this, the brains were pre-incubated in sample buffer at room temperature overnight. Each pre-incubated brain was placed in a sample cup containing choline acetyltransferase (ChAT) antibodies (AB144P, MilliporeSigma, United States), tyrosine hydroxylase (TH) antibodies (818001, Biolegend, United States), Lectin-DyLight™ 488 (DL1174-1, Vector laboratories, United States), Fab fragment donkey-anti-goat IgG (Alexa647, 705-607-003, Jackson Lab, United States), and Fab fragment donkey-anti-mouse IgG (Rhodamine X Red, 715-297-003, Jackson Lab, United States) diluted in sample buffer. The immuno-labeled brains were washed with PBST for 6 hours, post-fixed with 4% PFA at room temperature for two days, and then washed with PBS to remove residual PFA. The brains were then RI-matched with NFC1 and NFC2 solutions (Nebulum technology, Taiwan) before imaging.

Volumetric imaging was performed using a light-sheet microscope (SmartSPIM, LifeCanvas Technologies, South Korea) and 4x objective. 3D visualization was performed using Imaris software (Bitplane, United Kingdom). For angiogenesis evaluation, 3 regions of interest (ROIs) close to the injury were selected (each size of 1 cubic millimeter). The

surface function of Imaris software was used to segment the blood vessels and calculate the total volume and surface area. The length and bifurcation point numbers were calculated using Vessap.[7] Erosion and dilation were performed to remove false-negative pixels and avoid false centerline detections. Next, the centerlines were extracted with a 3D thinning algorithm.[8] The bifurcation points were detected using the surrounding pixels of each point to decide a point that splits into two or more vessels. For dopaminergic and cholinergic neuronal recovery evaluation, the volume of specific regions (striatum, nigrostriatal fiber tract, and fasciculus retroflexus) was calculated using Imaris software. The volume of certain regions of each group was normalized by the volume of corresponding contralateral regions. All statistical analyses were performed using GraphPad Prism (GraphPad Software Inc., United States).

Animal Behavior Experiment

There were three different behavioral tests in this study: cylinder test, grid test, pasta test.[9] Mice were tested on week 0, 1, 2, 3, 4, 5 post-injuries. The cylinder test: the purpose of the cylinder test was to measure the dexterity of their forelimbs. A Plexiglas cylinder was applied, and mice were placed inside it alone to observe their forelimbs. The forelimbs would touch the cylinder and the asymmetry of left and right forelimbs will be recorded. Afterwards, the asymmetry index could be calculated. The expression was: $\{[R + (R + L)] - [L + (R + L)]\} / \{[R + (R + L)] + [L + (R + L)]\}$. The design of Plexiglas cylinder was 15 cm in height with a diameter of 10 cm. The grid test: the purpose of this test was to measure the dexterity of their hindlimbs. A square wire fence (2.5×2.5 cm^2) with a grid area (length 12 cm, width 36 cm, height 10) was used in this test. Mice were placed on the fence alone. When they walked on the fence, they might step on the grid holes and miss their footing (foot-faults). The foot-faults could be calculated. The expression was: (the steps of foot-faults / the total steps) × 100 %. Pasta test: the purpose of this test was to measure the controllability of their forelimbs. The uncooked pasta (1 mm in diameter) was cut to 2.6 cm in length. During the test, the time a mouse spent to finish eating a piece of pasta was record.

Statistical Analysis

Statistical analysis quantitative data were presented as mean ± standard deviation (SD). Statistical comparisons were made using one-way ANOVA (for multiple comparisons) or unpaired Student's t-test (between two groups). The significance of differences was treated as follows: * represents $p < 0.05$ and ** represents $p < 0.01$. Statistical analysis was performed using GraphPad Prism Software Version 5.0.

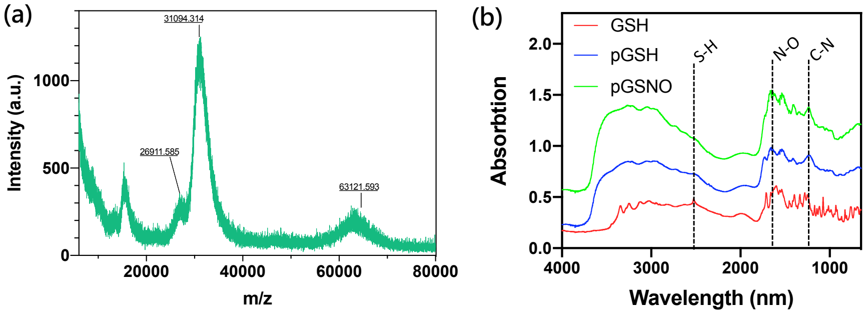

Figure S1. (a) The molecular weight of pGSNO was determined to be 31 kDa using MALDI-coupled time-of-flight mass spectrometry (MALDI-TOF MS). (b) The FTIR spectra of GSH, pGSH, and pGSNO. In pGSH, the presence of C-N bonding is evident at wavenumbers of 1255 and 1571 cm^{-1}. On the other hand, in pGSNO, the N-O bonding characteristic is observed at 1383 cm^{-1}, indicating the formation of S-NO bonds. S-H bonding signal is absent in pGSNO due to the replacement of S-H bonds by S-NO bonds.

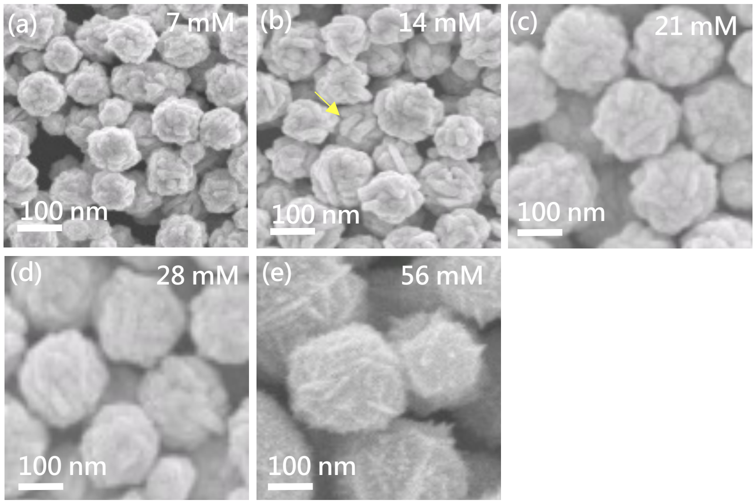

Figure S2. SEM images of GYs treated with various concentrations of hydroquinone (7 to 56 mM).

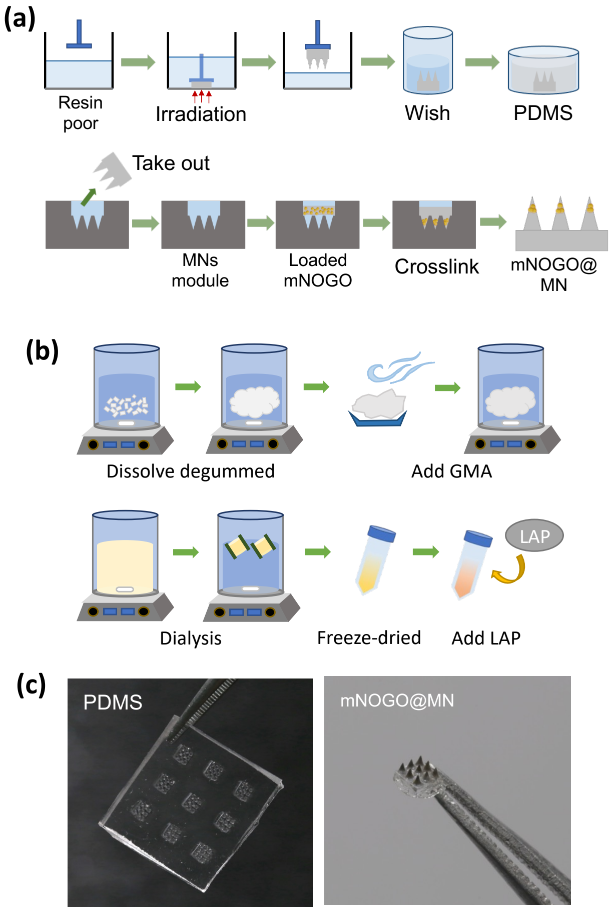

Figure S3. Preparation and characterization of conductive mNOGO@MN. (a) 3D printer printed out the resin MN, and the molds are established by PDMS. The materials (e.g., Sil-MA solution, drug) are filled into the molds and cured by UV light. The design of microneedles by SOLIDWORKS. (b) Modification of silk fibroin molecule with methacrylic anhydride (MA). Silk fibroin was fixed with MA covalently. (c) The images of 3D-printed PDMS molds and mNOGO@MN.

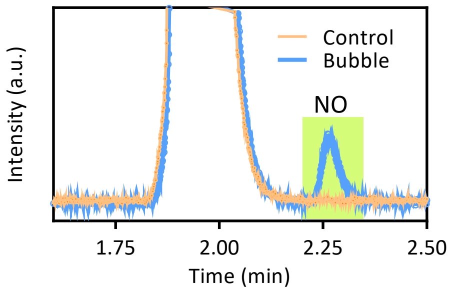

Figure S4. GC analysis of released bubbles from NOGO with HFMF treatment.

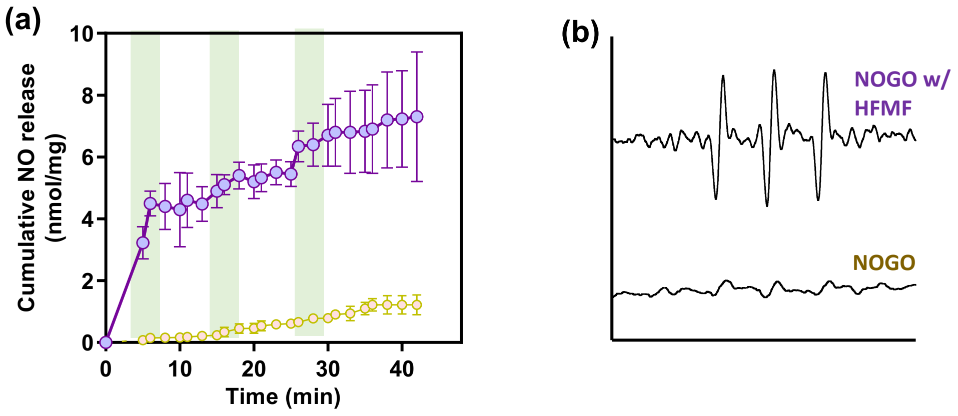

Figure S5. Cumulative NO release pattern of mNOGO detected by DAF-FM (4-Amino-5-Methylamino-2',7'-Difluorofluorescein) is a fluorescent probe commonly used to measure nitric oxide (NO) levels under intermittent application of HFMF (2.8 kW and 1 MHz) (n = 5). (b) ESR spectra of DMPO adducts produced from NOGO following treatment with HFMF.

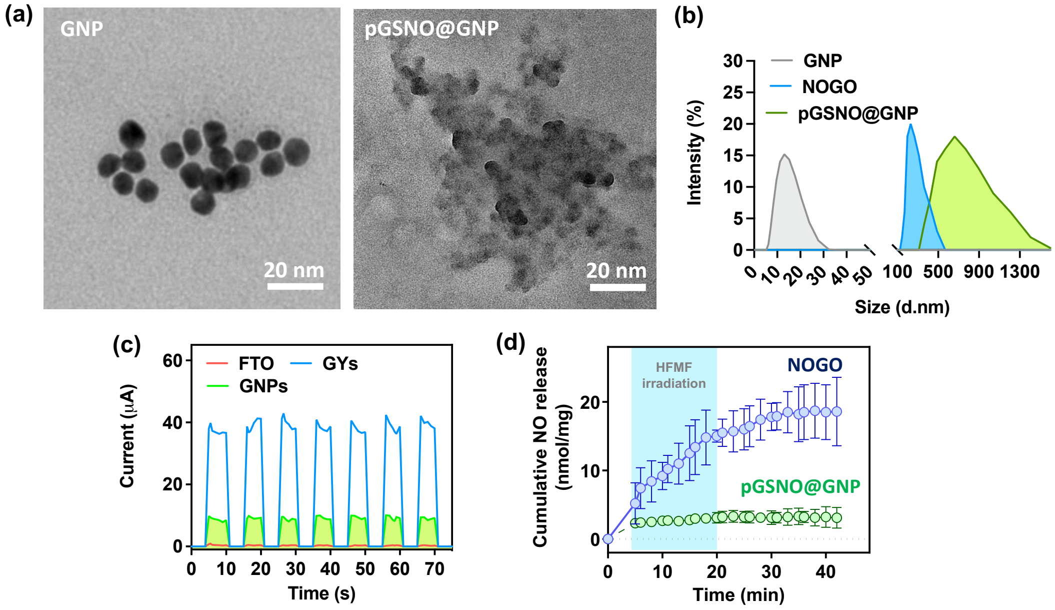

Figure S6. (a) TEM images of gold nanoparticles (GNPs) and pGSNO-loaded GNP (pGSNO@GNP). (b) Size distribution of GNP, NOGO and pGSNO@GNP. (c) Output currents on various materials generated by HFMF with on/off control. (d) The NO release from NOGO and pGSNO@GNP was measured after incubation in PBS at 37 °C with 15-minute HFMF treatment (blue area).

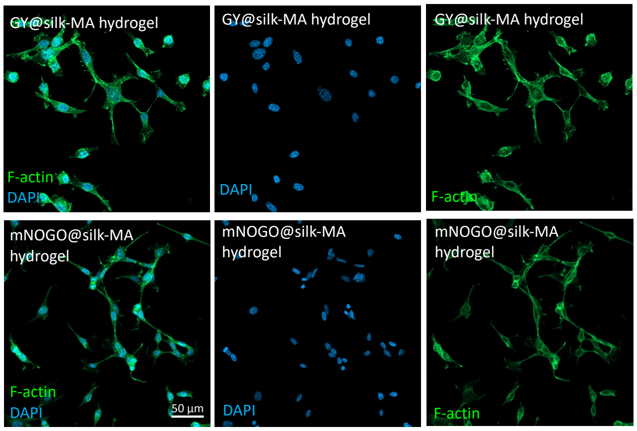

Figure S7. Confocal images of co-culture of NIT-3T3 cells and materials. Blue fluorescence represents nuclei staining with DAPI, and green fluorescence performs cytoskeleton staining with F-actin.

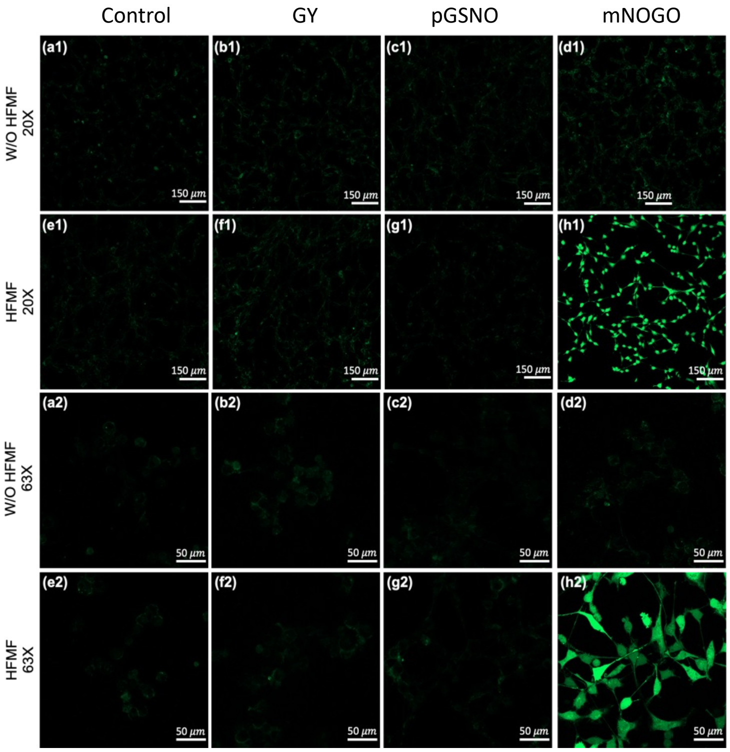

Figure S8. CLSM images of *in vitro* nitric oxide (NO) release. (a-d) DAF-FM fluorescence intensity of each group without high-frequency magnetic field (HFMF) stimulation. (e-f) DAF-FM fluorescence intensity of each group under HFMF stimulation. mNOGO group demonstrates a significant increase in green fluorescence when exposed to HFMF stimulation, suggesting that the nanoparticles produce NO.

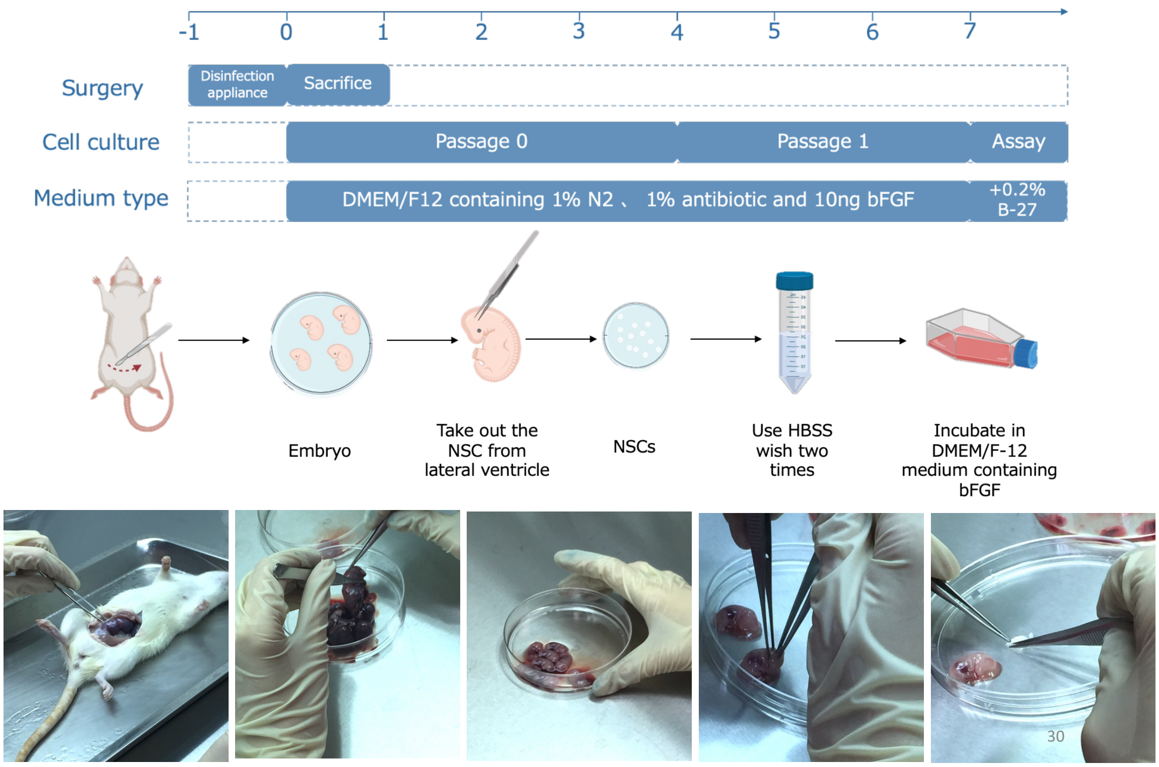

Figure S9. The primary neural stem cells extraction process.

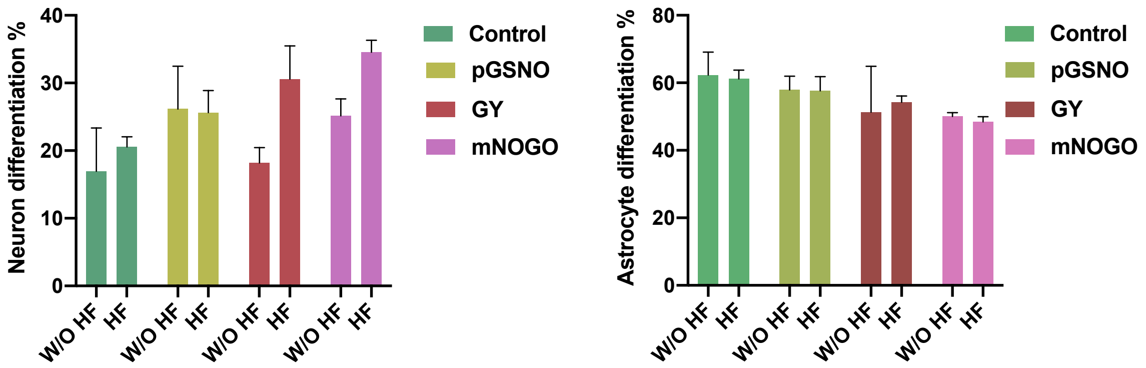

Figure S10. Quantitative analysis of confocal images, including neuron and astrocyte differentiation ratio.

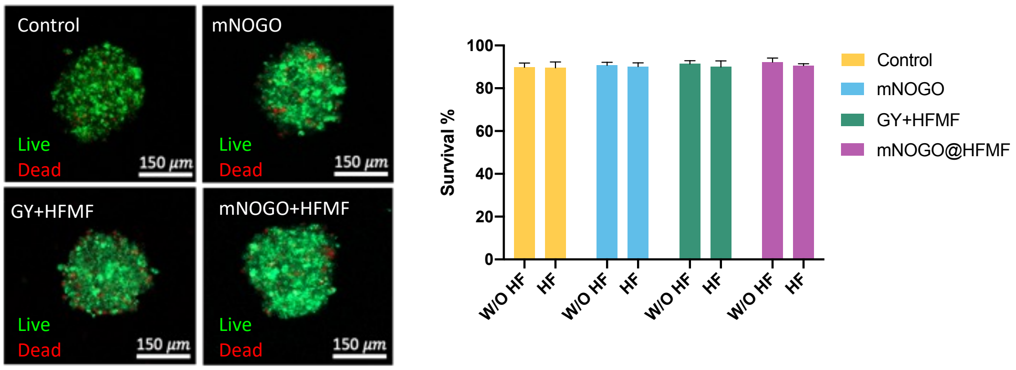

Figure S11. The Live/Dead assay was conducted using NSCs (neural stem cells) to assess their viability. The experimental groups, including mNOGO, GY+HFMF, and mNOGO+HFMF, were treated with or without HFMF. Importantly, all these groups demonstrated non-toxicity both in the presence and absence of HFMF treatments. To analyze the assay results, Image J software was employed for image analysis.

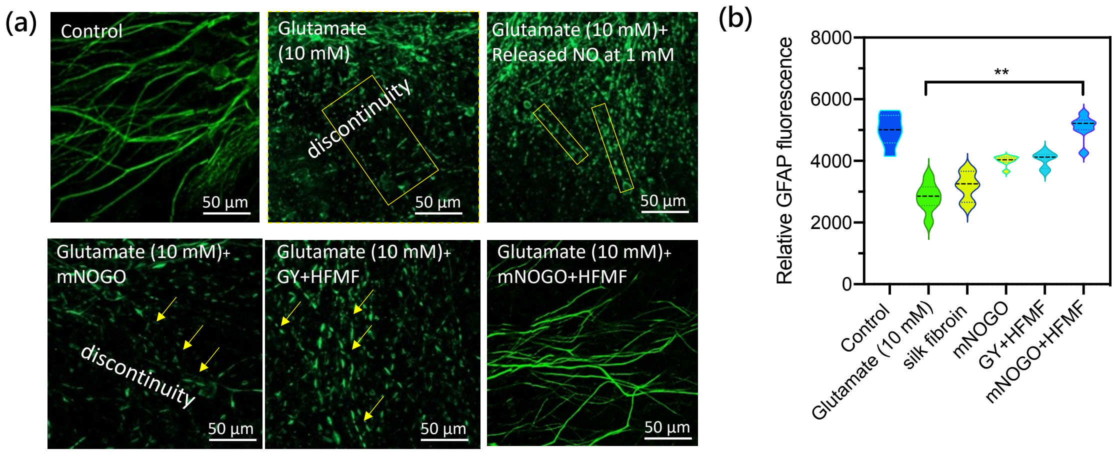

Figure S12. (a) CLSM images of NSCs after various treatments against glutamate-induced excitotoxicity under neuroprotection. (b) The relative fluorescence intensity of GFAP after various treatments.

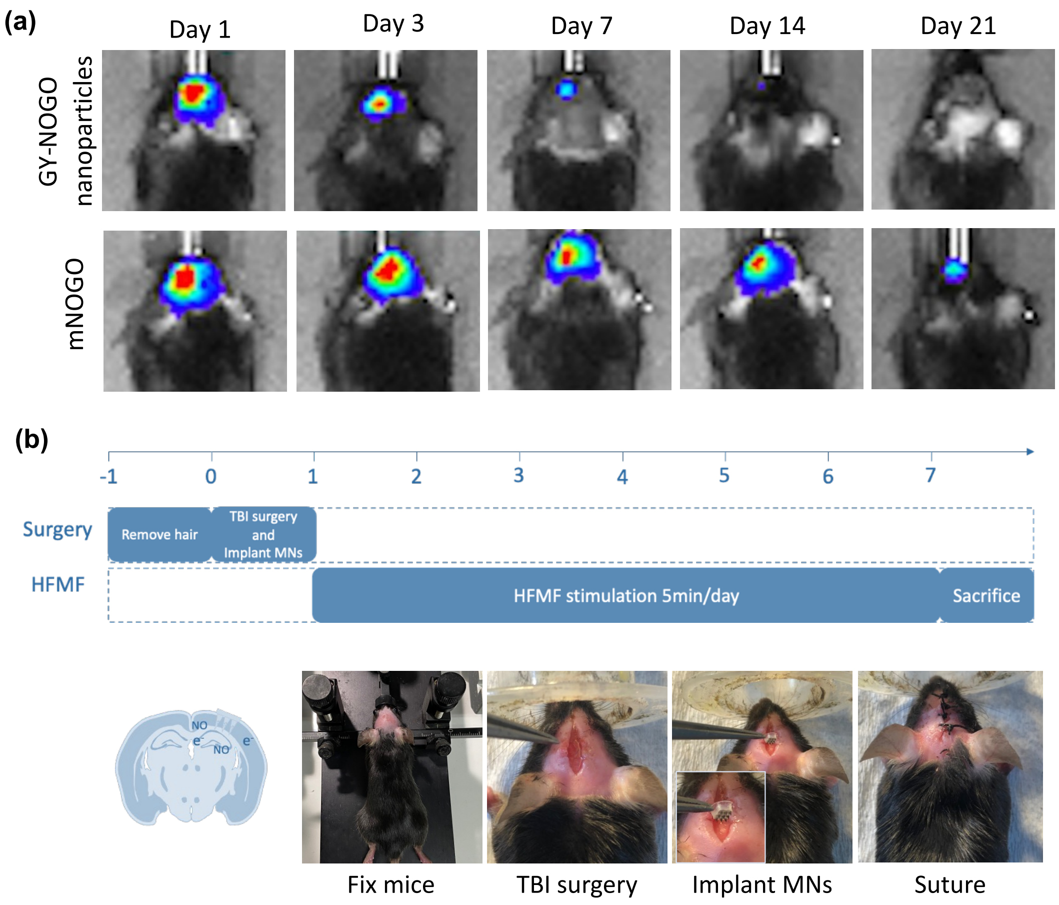

Figure S13. (a) An *in vivo* imaging system (IVIS, (200 Series, Caliper Life Science, U.S.A.) spectrum imaging system obtained fluorescence images after implanting GY-NOGO nanoparticles and mNOGO. The particles were labelled by Cy5.5. (Cy5.5 channel, excited wavelength = 675 nm). (b) The process of traumatic brain injury (TBI) surgery and treatments involves improving various aspects of the medical procedures and therapies aimed at managing and rehabilitating animals with TBI.

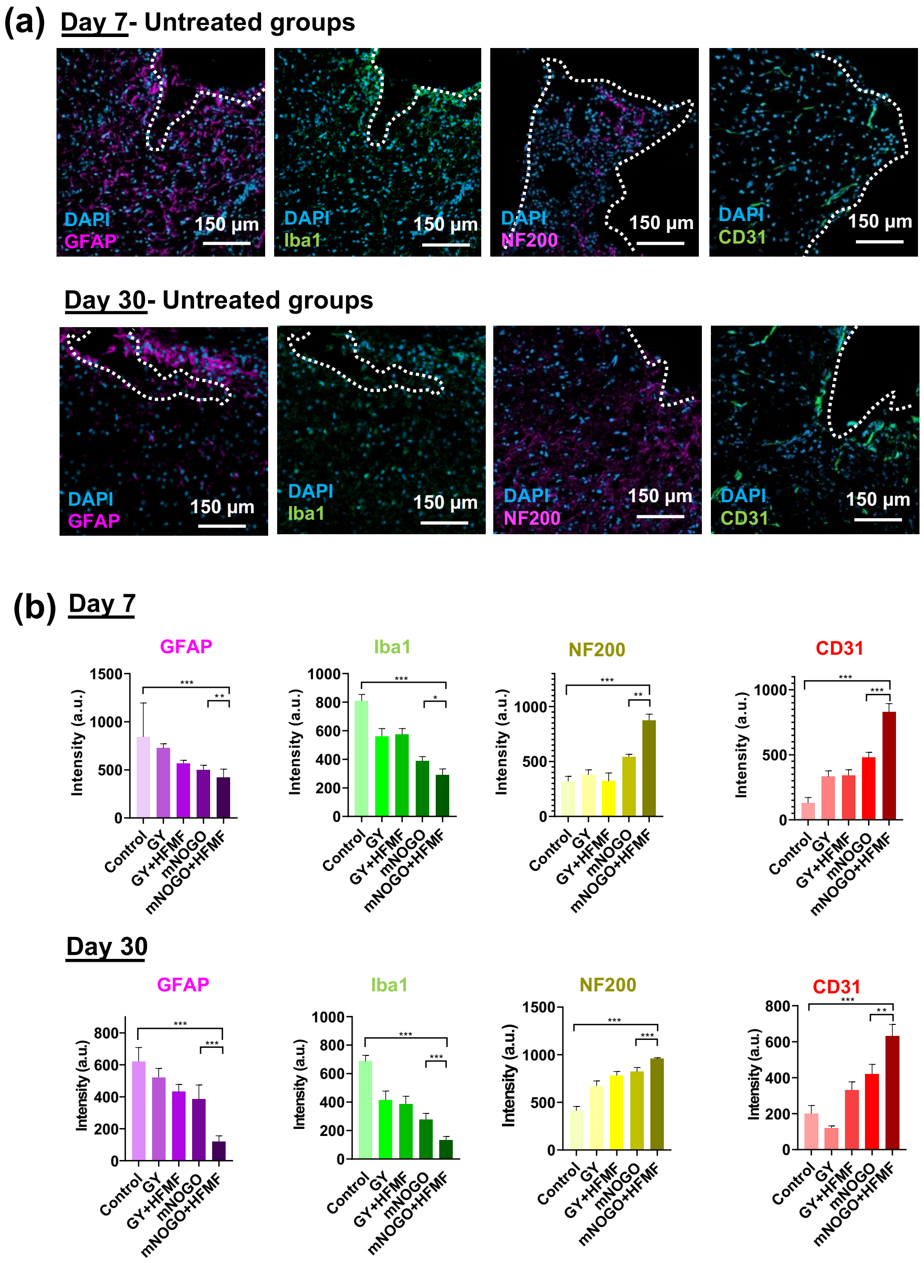

Figure S14. (a) Confocal laser scanning microscopy (CLSM) images of areas adjacent to the injury site, showing astrocytes (stained with GFAP), microglia/macrophages (green, stained with Iba1), neurofilament cells (NF200), and blood vessels (CD31) at 7- and 30-days post-injury, respectively. Blue fluorescence represents nuclei stained with DAPI. (b) The quantifications of fluorescence intensities for *in vivo* immunohistochemistry analysis.

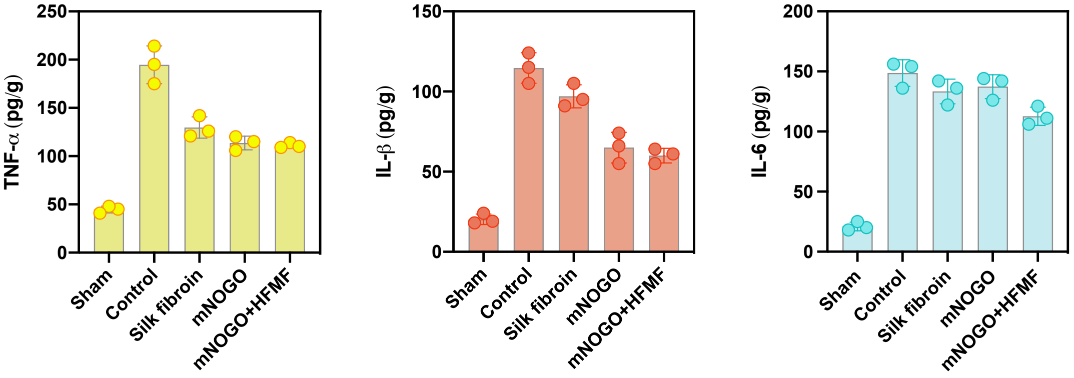

Figure S15. Quantification of immune factor concentrations, specifically TNF-α, IL-β, and IL-6, within brain tissues.

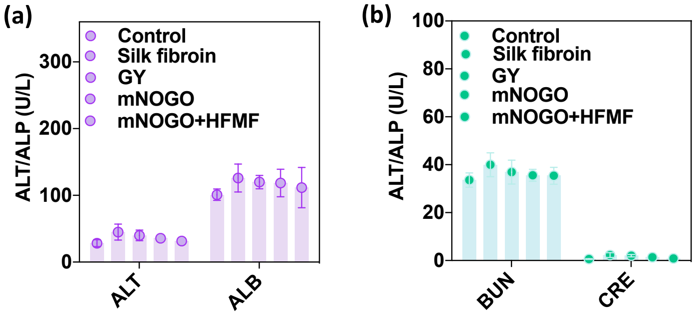

Figure S16. Biochemical indices of (a) hepatic and (b) renal parameters following a 72-hour treatment duration. (n = 3).

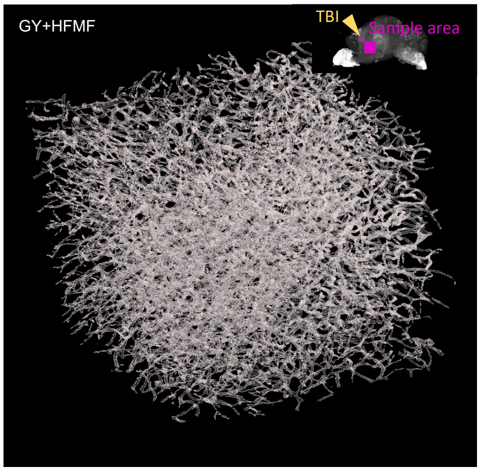

Figure S17. GY+HFMF brain images of lectin for vascular imaging at peri-trauma regions. The quantification of blood vessel volume, length, surface area, and vessel branches was shown in Fig. 5c.

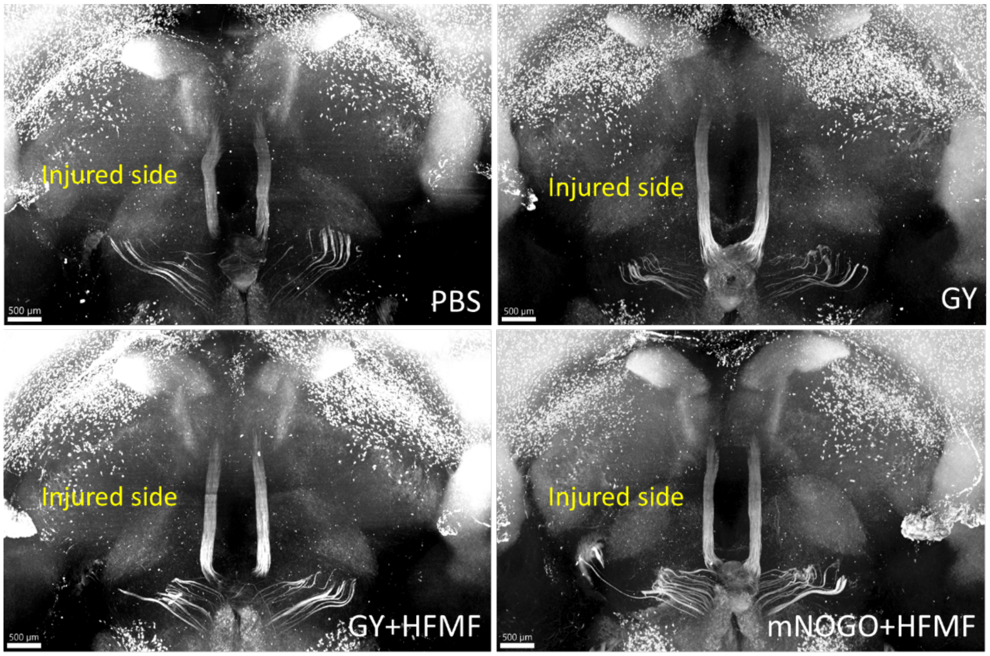

Figure S18. Brain images stained by Choline acetyltransferase (ChAT) that provides insight into the function of cholinergic neurons in different parts of the nervous system.

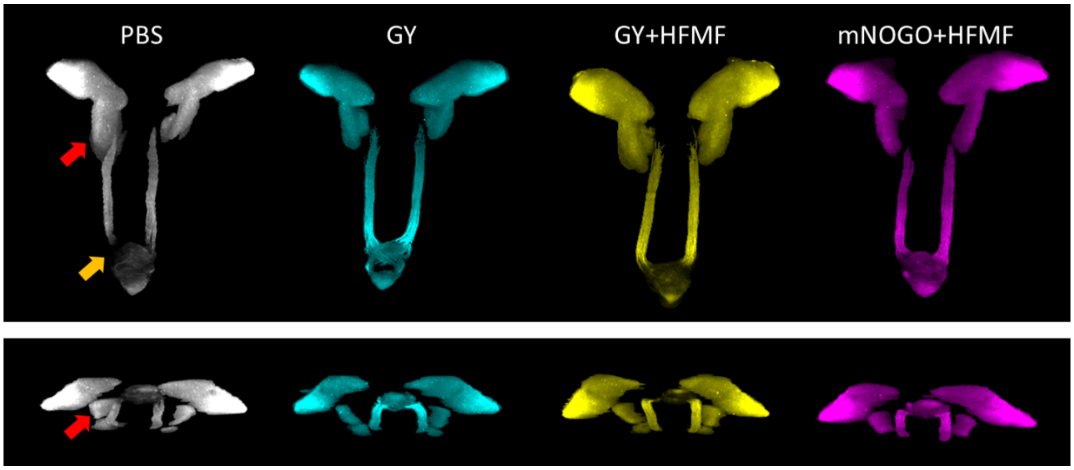

Figure S19. Enhanced images of Hbn-IPN complex. Horizontal view (upper row) and coronal view (bottom row) are shown. The enlarged Hbn (red arrows) and reduced FR (orange arrow) are indicated.

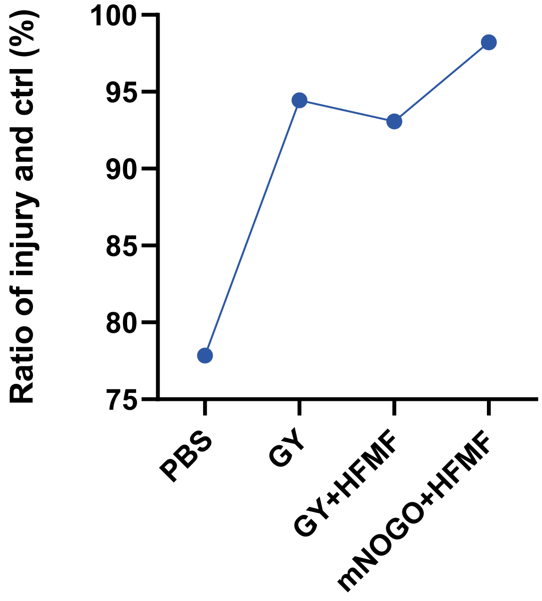

Figure S20. The relative volume of FR of the control (PBS), GY, GY+HFMF and mNOGO+HFMF mice.

References

[1] W. Cheng, Y. L. Su, H. H. Hsu, Y. H. Lin, L. A. Chu, W. C. Huang, Y. J. Lu, C. S. Chiang, S. H. Hu, *ACS nano* **2022,** *16,* 4028.

[2] S. W. Park, T. E. Kim, Y. K. Jung, *Analytica Chimica Acta* **2021,** *1165,* 338513.

[3] J. H. Fang, H. H. Hsu, R. S. Hsu, C. K. Peng, Y. J. Lu, Y. Y. Chen, S. Y. Chen, S. H. Hu, *NPG Asia Mater.* **2020,** *12,* 61.

[4] L. Váradi, M. Breedon, F. F. Chen, A. Trinchi, I. S. Cole, G. Wei, *RSC Adv.* **2019,** *9,* 3994.

[5] Y. G. Park, et al. *Nat. Biotechnol.* **2019,** *37,* 73.

[6] D. H. Yun, et al. *bioRxiv* **2019,** 660373. doi:10.1101/660373.

[7] M. I. Todorov, et al. *Nat. Methods* **2020,** *17,* 442.

[8] T. C. Lee, R. L. Kashyap, C. N. Chu, *CVGIP Graph. Model. Image Process.* **1994,** *56,* 462.

[9] L. R. Nih, S. Gojgini, S. T. Carmichael, T. Segura, *Nat. Mater.* **2018,** *17,* 642.
